# Update on the Pathomechanism, Diagnosis, and Treatment Options for Rheumatoid Arthritis

**DOI:** 10.3390/cells9040880

**Published:** 2020-04-03

**Authors:** Yen-Ju Lin, Martina Anzaghe, Stefan Schülke

**Affiliations:** 1 Paul-Ehrlich-Institut, Vice President’s Research Group 1: Molecular Allergology, 63225 Langen (Hesse), Germany; Yen-Ju.Lin@pei.de; 2 Paul-Ehrlich-Institut, Product Testing of Immunological Biomedicines, 63225 Langen (Hesse), Germany; Martina.Anzaghe@pei.de

**Keywords:** rheumatoid arthritis, autoimmunity, TNF, IL-6

## Abstract

Rheumatoid arthritis (RA) is an autoimmune disease that involves multiple joints bilaterally. It is characterized by an inflammation of the tendon (tenosynovitis) resulting in both cartilage destruction and bone erosion. While until the 1990s RA frequently resulted in disability, inability to work, and increased mortality, newer treatment options have made RA a manageable disease. Here, great progress has been made in the development of disease-modifying anti-rheumatic drugs (DMARDs) which target inflammation and thereby prevent further joint damage. The available DMARDs are subdivided into (1) conventional synthetic DMARDs (methotrexate, hydrochloroquine, and sulfadiazine), (2) targeted synthetic DMARDs (pan-JAK- and JAK1/2-inhibitors), and (3) biologic DMARDs (tumor necrosis factor (TNF)-α inhibitors, TNF-receptor (R) inhibitors, IL-6 inhibitors, IL-6R inhibitors, B cell depleting antibodies, and inhibitors of co-stimulatory molecules). While DMARDs have repeatedly demonstrated the potential to greatly improve disease symptoms and prevent disease progression in RA patients, they are associated with considerable side-effects and high financial costs. This review summarizes our current understanding of the underlying pathomechanism, diagnosis of RA, as well as the mode of action, clinical benefits, and side-effects of the currently available DMARDs.

## 1. Introduction

Rheumatoid arthritis (RA) is a chronic autoimmune disease affecting the joints. It is characterized by a progressive symmetric inflammation of affected joints resulting in cartilage destruction, bone erosion, and disability [1]. While initially only a few joints are affected, in later stages many joints are affected and extraarticular symptoms are common (see below) [2].

With a prevalence ranging from 0.4% to 1.3% of the population depending on both sex (women are affected two to three times more often than men), age (frequency of new RA diagnoses peaks in the sixth decade of life), and studied patient collective (RA frequency increases from south to north and is higher in urban than rural areas) [1,2,3,4,5], RA is one of the most prevalent chronic inflammatory diseases [1].

Clinically, the symptoms of RA significantly differ between early stage RA and insufficiently treated later stages of the disease. Early stage RA is characterized by generalized disease symptoms such as fatigue, flu-like feeling, swollen and tender joints, and morning stiffness; and is paralleled by elevated levels of C-reactive protein (CRP) and an increased erythrocyte sedimentation rate (ESR) [6]. In contrast, insufficiently treated RA displays a complex clinical picture with the occurrence of serious systemic manifestations such as pleural effusions, lung nodules and interstitial lung disease, lymphomas, vasculitis in small or medium-sized arteries, keratoconjunctivitis, atherosclerosis, hematologic abnormalities (e.g., anemia, leukopenia, neutropenia, eosinophilia, thrombocytopenia, or thrombocytosis), joint malalignment, loss of range of motion, bone erosion, cartilage destruction, and rheumatic nodules (in detail reviewed in [1,2,7]). Taken together, these systemic manifestations caused by the chronic inflammatory state in RA patients result in an increased mortality.

## 2. Development of Rheumatoid Arthritis

**Figure 1:**
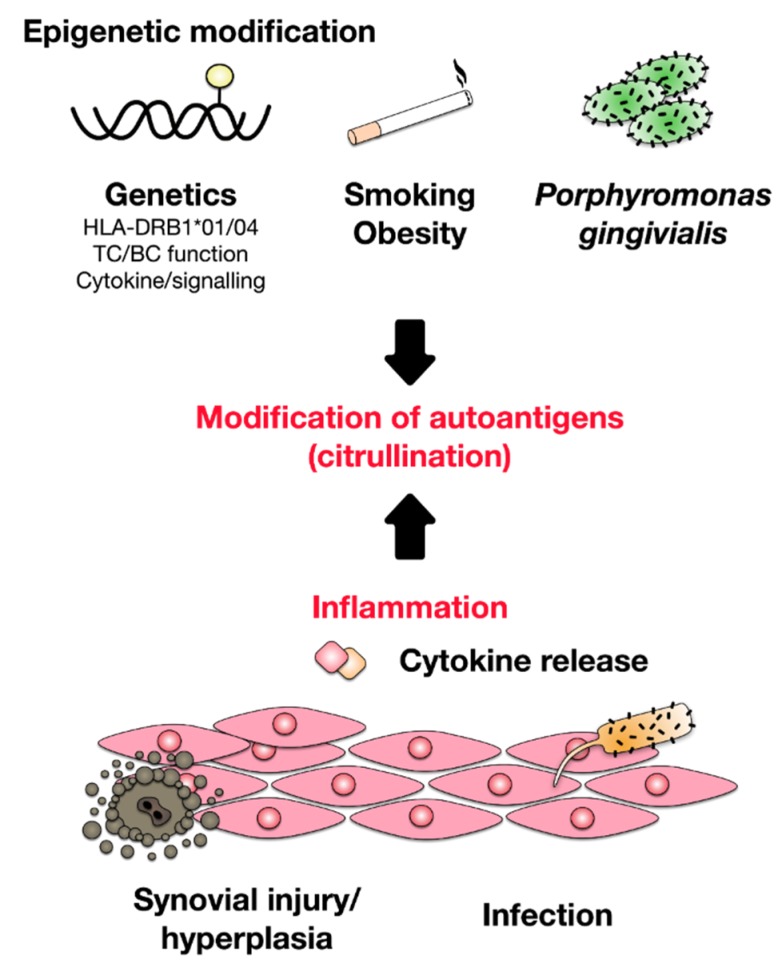
Factors contributing to rheumatoid arthritis (RA) development. Both environmental (smoking, obesity, as well as infections with certain pathogens such as *Porphyromonas gingivialis*) and genetic factors (epigenetic modifications, genetic polymorphisms influencing antigen presentation (e.g., the HLA genes HLA-DRB1*01/04), T- and B cell function, cytokine production, and signal transduction following immune cell activation) contribute to the development of RA. Moreover, also synovial injury and hyperplasia of synovial fibroblasts can contribute to the establishment of RA via the triggering of inflammatory conditions. Overall, these processes lead to the modification of autoantigens (mostly by citrullination) which generates neoepitopes by a loss of surface charge and an increased susceptibility to proteolytic degradation.

While the cause of RA is unknown, both genetic and environmental factors were shown to contribute to RA development [8] (Figure 1). As it is hypothesized for other autoimmune diseases, it is likely that the initial establishment of RA requires two separate events: (1) genetic predisposition of the respective patient resulting in the generation of autoreactive T and B cells, and (2) a triggering event, such as viral and bacterial infections or tissue injury, providing the activated Antigen-presenting cells (APCs) to activate the previously generated autoreactive lymphocytes, resulting in disrupted tolerance and subsequent tissue/organ destruction. Therefore, RA likely develops in genetically predisposed individuals due to a combination of genetic variation, epigenetic modification, and environmental factors initiated by a stochastic event (e.g., injury or infection) [1]. Risk factors for the development of RA were reported to include smoking, obesity, exposition to UV-light, sex hormones, drugs, changes in microbiome of the gut, mouth, and lung, periodontal disease (periodontitis), and infections [1,2,5,7,9,10]. Among these factors, the link between periodontal diseases and RA development is especially interesting.

While the association between periodontitis and RA development was recognized as early as the 19th century [11], recent studies have demonstrated that infections with the common periodontal bacterium *Porphyromonas gingivalis* can result in the induction of autoimmune responses via the citrullination of host peptides [2,9]. During this process, which is catalyzed by the enzyme protein arginine deiminase (PAD), positively charged arginine residues of “self” proteins are converted into neutral citrulline residues, resulting in a net loss of surface charge, an increased susceptibility of the citrullinated “self” proteins to protein degradation, and the generation of neoepitopes [2,9].

This breach of local tolerance by *P. gingivalis* expressing PADi4 (facilitating the conversion of arginine to citrulline) promotes autoimmune responses as well as the downstream generation of anti-citrullinated protein antibodies (ACPAs) [12]. In addition, other viral (Epstein–Barr virus) and bacterial infections (*Proteus mirablis, Escherichia coli*) were suggested to trigger the development of RA by mechanisms of molecular mimicry caused by similarities between amino acid sequences of “self” antigens and certain bacterial- or viral proteins [1,7,13,14,15].

Besides citrullination, carbamylation of lysine residues also contributes to the generation of neoepitopes from several “self” proteins (e.g., collagen, fibrinogen, or vimentin) and the subsequent breaking of immunological “self” tolerance [7,16].

Since both a family history of RA increases the risk to develop RA three to five times and concordance risk rates in identical twins are increased compared to both non-related control collectives and non-identical twins, we have to assume that genetic factors also contribute to RA development [1,17].

Genome wide association studies using single nucleotide polymorphisms (SNPs) have suggested more than 100 loci to be associated with RA development [1,18]. As expected, many of these loci are involved in the induction, regulation, and maintenance of immune responses and are shared with other chronic inflammatory diseases. Some of the RA risk factors are the presence of certain HLA alleles [19], alterations in co-stimulatory pathways (e.g., via changes in CD28 or CD40 expression) [20], as well as changes in innate immune cell activation [21], lymphocyte activation thresholds (e.g., PTPN22) [22], or cytokine signaling [1]. Among the genes contributing to RA development, HLA-DRB1 alleles (DRB1*01 and DRB1*04; DQ8) account for approximately 50% of the observed genetic susceptibility [2,23,24]. Studies have suggested that these HLA alleles, which share amino acid sequences within their peptide binding grooves, are able to preferentially present certain peptide epitopes derived from relevant RA autoantigens [1,25]. In addition, some of these HLA-DRB1 alleles are associated with more aggressive bone erosion and increased mortality rates [26].

Taken together, these findings suggest a strong T cell-dependent component in RA and indeed Th1 and Th17 T cell subsets are the predominant cell types found in the inflamed synovial tissue (see also below “pathomechanism of RA”) [2,27].

## 3. Pathomechanism of RA

In RA autoimmune tissue destruction presents as synovitis, an inflammation of the joint capsule consisting of the synovial membrane, synovial fluid, and the respective bones [7]. This joint inflammation is initiated and maintained by a complex interplay between different dendritic cell (DC) subtypes, T cells, macrophages, B cells, neutrophils, fibroblasts, and osteoclasts. Since the ubiquitously present RA-specific autoantigens cannot be completely cleared, this continuous immune cell activation results in a self-perpetuating, chronic inflammatory state in the joint and swelling of the synovial membrane that is recognized by the affected patients as pain and joint swelling [1]. This chronic inflammatory milieu in the arthritic joint in turn leads to an expansion of the synovial membrane termed “pannus” which invades the periarticular bone at the cartilage–bone junction, resulting in bone erosion and cartilage degradation [7].

### 3.1. Contribution of Dendritic Cells to Establishment and Maintenance of Inflammation in RA

DCs have a critical function in regulating immune responses by taking up, processing, and presenting antigens to naïve T cells. In this context, the DC phenotype, characterized by the expression of surface molecules and the production of both cytokines and chemokines, determines the balance between either immune system activation or the induction and maintenance of tolerance.

Accumulating evidence suggests that both an altered distribution and function of DCs in RA as well as other autoimmune diseases contribute to autoimmune inflammation (reviewed in [28]). In this context, a reduced frequency of both conventional DCs and plasmacytoid DCs in the plasma of RA patients was reported [29], likely caused by an enhanced migration of DCs to the inflamed joint [28]. This DC recruitment was hypothesized to be mediated by an increased expression of CCR6 on the DCs, with CCR6 being the receptor for the chemokine CCL20 which is highly expressed in synovial tissue [30].

Once attracted to the joint, mature DCs were shown to produce the cytokines IL-12 and IL-23 which promote antigen-specific Th17 responses, resulting in imbalances between Th1-, Th2-, and Th17 responses [31,32,33]. In this context, especially CD14^+^CD1a^+^CD1c^+^ inflammatory DCs (locally differentiating from monocytes invading the inflamed joint) in synovial fluid were suggested to play an important role in the pathogenesis of RA by effectively activating Th17 cells in RA joints via their production of TGF-β, IL-1β, IL-6, and IL-23 [31].

Moreover, activated plasmacytoid dendritic cells (pDCs) were also reported to contribute to overall inflammation in RA patients via the secretion of IFN-α, IFN-β, IL-18, and IL-23. In addition, pDCs may promote the production of autoantibodies (see below) via the expression of anti-apoptotic B cell activating factor (BAFF) [32]. In line with this, ACPA seropositive RA patients have been shown to have higher numbers of pDCs in the synovium than their ACPA negative counterparts [32]. RA patients also show an increased transcriptional activity of interferon-stimulated genes. Thus, IFNs might have an important role both in the initial loss of “self” tolerance as well as in the chronic, established phase of RA [34,35].

Therefore, enhanced pro-inflammatory cytokine production in conjunction with the activated status of DCs may promote the presentation of autoantigens to T cells and the perpetuation of inflammatory responses. In addition to changes in cytokine secretion, DC activation in the inflammatory milieu of the synovium also modulates the expression pattern of e.g., chemokine receptors regulating DC migration. For example, DCs in RA synovium were shown to express lower levels of CCR7, resulting in reduced emigration rates of mature DC from inflamed tissues and the maintenance of local inflammation [30,36].

### 3.2. Joint Inflammation in RA is Mediated by T cells, B cells, Macrophages, and Fibroblasts

Initiated by both epithelial cells in the synovium and activated antigen presenting cells priming autoantigen-specific T- and B cell responses in both lymph nodes and local tissues, the main infiltrating cells in the affected joints are T cells, B cells, and monocytes [7].

Activated T cells migrating to the synovium locally interact with resident macrophages, dendritic cells, synoviocytes, and osteoclasts. Here, several T cell subsets and their complex interactions likely contribute to RA pathology (reviewed in [37]).

Via their secretion of IL-2, IFN-γ, and TNF-β, Th1 cells potently provide help to other immune cells, resulting in the activation of macrophages and B cells, thereby initiating and perpetuating inflammatory responses in the synovium [37,38,39]. In addition to their helper function in RA inflammation, CD4^+^CD28^null^ cells co-expressing perforin and granzymes, molecules more commonly found in CD8^+^ cytotoxic T cells, were recently shown to be increased in peripheral blood of a subset of RA patients [40,41,42]. Moreover, CD4^+^ cells expressing perforin have been observed in synovial fluid and tissue [42,43,44], suggesting that these cells might contribute to tissue damage and the maintenance of inflammation in RA patients.

In addition, an increased frequency of CXCR5^+^ICOS^+^CD4^+^ T follicular helper cells correlating with both ACPA titers and overall disease severity was reported in peripheral blood of RA patients [45,46,47]. However, currently, their contribution to RA pathology is unclear.

Th17 cells, induced by the cytokines IL-6, IL-1β, IL-21, TGF-β, and IL-23 present in synovial joints [48,49], were shown to recruit neutrophils, activate B cells, and promote osteoclastogenesis [50,51]. However, the role of IL-17A in RA is up for debate, since therapeutic targeting of either IL-17A or the IL-17R showed lower efficacy than for example in the treatment of psoriasis [52,53]. Moreover, anti-TNF-α treatment was shown to trigger IL-10 production from human Th17 cells [54], suggesting that Th17 cells could also have immunosuppressive capacities in RA. Therefore, it was suggested, that Th17 cells may be important during early stages of the disease, while in later stages Th1 cells differentiated into cytotoxic CD4^+^ T cells may drive both direct tissue damage and pro-inflammatory cytokine production [37].

Moreover, the delicate balance between effector and regulatory T (Treg) cell subsets is likely to influence both disease establishment and progression. Here, studies suggest that the inflammatory milieu in RA patients may contribute to (1) Treg dysfunction preventing the control of autoreactive T cells and (2) differentiation of Tregs into pathologic T cells. In line with this hypothesis, CD4^+^CD25^+^Foxp3^+^ Tregs with the potential to convert into pathogenic Th17 cells were shown to accumulate in inflamed synovium [55,56]. Moreover, Tregs in RA patients were shown to locally lose their suppressive capacity in synovial fluid while Tregs in peripheral blood retained their suppressive properties [57]. Finally, a distinct population of Tregs with TGF-β-dependent suppressive capacity could be induced by inhibition of TNF-α [58,59].

### 3.3. Contribution of Cytokines to Inflammation in RA

**Figure 2:**
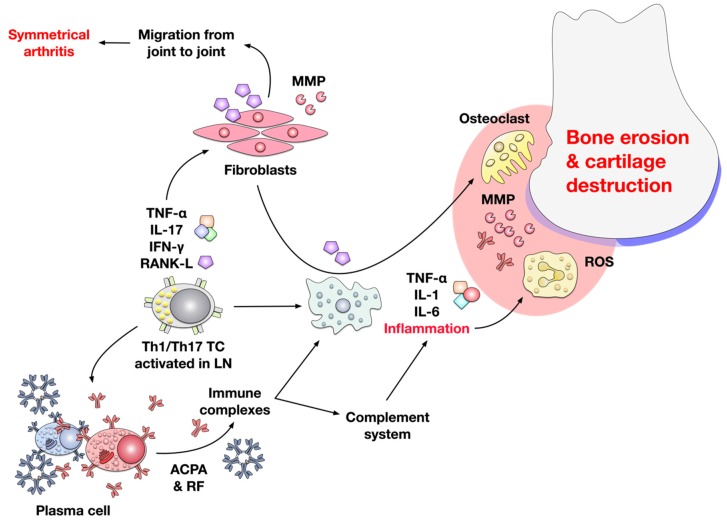
Pathomechanism of RA. Inflammation in RA is induced by autoreactive Th1- or Th17 T cells primed in the lymph nodes (LN) or locally by activated Antigen-presenting cells (APCs) that present autoantigen-derived peptides. In the affected joint, activated autoreactive T cells subsequently activate macrophages and fibroblasts via the secretion of the pro-inflammatory mediators TNF-α, IL-17, IFN-γ, and receptor activator of nuclear factor KB ligand (RANK-L). Activated macrophages in turn secrete large amounts of the strongly pro-inflammatory cytokines TNF-α, IL-1β, and IL-6 which promote the establishment and maintenance of an inflammatory milieu in the synovium. Activated T cells also provide help to autoreactive B cells resulting in the production of anti-citrullinated protein antibodies (ACPAs) and rheumatoid factor (RF) autoantibodies. These autoantibodies further drive inflammation by either direct macrophage activation of triggering the complement cascade. In addition, RANK-L produced by the activated fibroblasts promotes the differentiation of osteoclasts from macrophages. Together with fibroblast-derived matrix metalloproteases (MMPs), osteoclasts, and antibodies, activated neutrophils mediate inflammation-dependent cartilage destruction and bone erosion.

As signaling molecules both among immune cells as well as between immune- and tissue cells, cytokines have an important function in the establishment of RA inflammation. The main effector cytokines produced by infiltrating T cells are tumor necrosis factor (TNF)-α, IL-17A, interferon (IFN)-γ, and receptor activator of nuclear factor KB ligand (RANK-L) [1] (Figure 2).

Especially TNF-α, which is also produced by synovial macrophages, B-, and NK-cells is one of the most important mediators of joint inflammation in RA [60]. It is present in most arthritic biopsies and its overexpression induces spontaneous inflammation in various rodent arthritis models [61]. Early in vitro studies demonstrated that TNF-α could induce both cartilage degradation [62] and bone resorption [63]. Recently, TNF-α was also shown to enhance RANK-L secretion by osteocytes which further promotes osteoclastogenesis [64]. Interestingly, some studies pointed out that TNF-α can also directly induce the differentiation of monocyte/macrophage lineage cells into osteoclasts by a RANK-L-independent mechanism [65,66,67]. The other important role of TNF-α in the pathogenesis of RA is its ability to induce the production of other inflammatory cytokines, such as IL-1β and IL-6, which attract leukocytes and promote the establishment of an inflammatory milieu in the synovium [60].

IL-17A produced by Th17 cells promotes both the production of the pro-inflammatory cytokines IL-6, IL-8, and GM-CSF from epithelial, endothelial, and fibroblastic cells [68] and neutrophil recruitment [69], which leads to local inflammation and promotes disease progression. By these actions, IL-17A contributes to bone erosion, cartilage destruction, and neoangiogenesis in RA patients [70]. IL-17A triggers differentiation of osteoclast progenitors into mature osteoclasts and promotes RANK-L production by osteoblasts and synoviocytes, resulting in both reduced bone formation and enhanced bone erosion [71,72,73]. In addition, IL-17A was shown to also promote matrix metalloproteinase (MMP)-1 production by synoviocytes, leading to cartilage destruction [74]. Angiogenesis plays a critical role in the pathogenesis of RA (see Section 3.5). In this context, IL-17A was shown to enhance both endothelial cell migration [75] and the production of vascular endothelial growth factor (VEGF) by synovial fibroblasts [76].

Another important cytokine in the pathology of RA is IFN-γ. RA patients have high levels of IFN-γ in plasma, synovial tissue, and synovial fluid [77,78]. IFN-γ is produced by T cells, B cells, NK cells, monocytes/macrophages, DCs, and neutrophil granulocytes [79,80,81,82,83]. It binds to the ubiquitously expressed IFN-γ receptor where it triggers the activation of IFN-stimulated genes via multiple pathways including the Janus activated kinase-signal transducer and activator of transcription 1 (JAK–STAT1) pathway as well as the mitogen activated protein (MAP) kinase-, phosphatidylinositol 3-kinase (PI3K)-, and nuclear factor kappa-light-chain-enhancer of activated B cells (NF-κB)-pathways [84,85,86]. By these actions, IFN-γ enhances antigen presentation and macrophage activation [86]. IFN-γ-activated macrophages and monocytes in turn produce the chemokine CXCL10 which promotes differentiation of osteoclasts by inducing RANK-L and TNF-α secretion from CD4^+^ T cells [87,88]. Moreover, B cell-derived IFN-γ was shown to inhibit Treg differentiation in a murine model of proteoglycan-induced arthritis, thereby further promoting autoimmune responses [82].

Therefore, IFN-γ contributes to the establishment of early inflammation in RA by the above discussed mechanisms. However, it was also suggested to have tissue-protective effects in later stages of the disease by inhibiting for example RANK–RANK-L-mediated osteoclastogenesis, neutrophil influx, TNF-α-dependent synoviocyte proliferation, production of degradative enzymes, release of prostaglandin E2 and granulocyte-macrophage colony-stimulating factor (GM-CSF) (reviewed in [86,89]).

RANK-L, a member of the TNF superfamily, is an important regulator of bone regeneration and remodeling [90]. RANK-L binds to RANK and induces osteoclastogenesis [90]. Under normal physiological conditions, RANK-L is mainly produced by osteoblasts. However, in RA joints, immune cells (Th17 cells, macrophages, DCs, and activated B cells) [91,92] and fibroblast-like synoviocytes [93] are the main source of RANK-L. In RA patients, RANK-L induces an abnormal activation of osteoclasts, resulting in bone destruction [93]. In line with this, RANK-L knockout mice were protected from serum transfer-induced arthritis [94].

Macrophages activated in the pro-inflammatory milieu of the inflamed synovium in turn produce additional pro-inflammatory cytokines (IL-1β, IL-6, TNF-α) that contribute to increased inflammation by recruiting and activating other innate immune cells (e.g., neutrophils) to the site of synovitis. Activated neutrophils subsequently release high levels of oxidants, cytokines, and inflammatory agents including TNF-α, proteases, phospholipases, defensins, and myeloperoxidases at the site of RA in affected joints which further contribute to joint destruction [95,96].

Moreover, the pro-inflammatory cytokines IL-1β, IL-6, and TNF-α also both initiate and perpetuate the production of further degradative enzymes (e.g., MMPs) [97,98] and prostaglandins [62]. In addition, RANK-L produced by cytokine-activated fibroblasts in combination with TNF-α and IL-6 from activated immune cells induces the differentiation of macrophages and preosteoclasts into osteoclasts that are specialized in the degradation of bone material [1,7,99].

Activated fibroblasts not only produce RANK-L and MMPs directly contributing to local joint damage but also migrate between joints, promoting inflammation at other joints (explaining the symmetrical character of the disease) [100]. Therefore, besides activation of resident and infiltrating immune cells, joint inflammation in RA is also characterized by a specific tissue response in which local fibroblasts assume an aggressive pro-inflammatory phenotype characterized by matrix regulatory, osteoclast-generating, and invasive properties [101,102]. 

### 3.4. Contribution of B Cells and Autoantibodies to the Pathogenesis of RA

Antibodies, resulting from aberrant activation of autoreactive B cells, also significantly contribute to the pathology of RA by immune complex formation and subsequent complement activation [103]. Here, the two main types of autoantibodies found in RA are rheumatoid factor (RF) and anti-citrullinated protein antibodies (ACPAs). The presence of these two autoantibodies defines a patient as having “seropositive” RA [17]. While the pathology of RA can be heterogenous, the presence of RF and ACPA autoantibodies was correlated to aggravated disease symptoms, joint damage, and increased mortality [1].

RF autoantibodies are pentameric IgM antibodies that bind to the Fc portion of human immunoglobulin G (IgG). RF is found in 69% of patients with RA and has a specificity of 60%–85% for the disease [104,105]. Of note, RF can also be detected both in other disease states (e.g., infections, certain types of cancer, and other rheumatic diseases) as well as in healthy patients [106].

ACPA autoantibodies, which can be either of the IgG-, lgA-, or lgM-isotype, can bind to citrullinated protein residues of many “self” proteins such as for example type II collagen, histones, fibrinogen, fibronectin, vimentin, and α-enolase [107,108]. Similar to RF, ACPAs are found in 60–80% of RA patients, but their specificity for the disease is up to 85–99% [109]. The risk of onset of disease in patients positive for RF and ACPAs is 40% [110].

Interestingly, in a condition called pre-rheumatoid arthritis, ACPAs can be detected in the blood circulation of patients up to 10 years before the patients experience the first disease symptoms, making the presence of these antibodies a highly valuable tool for the diagnosis of early disease stages. Both ACPA concentrations and epitope diversity increase alongside the concentration of pro-inflammatory cytokines over the course of the disease and ACPAs were shown to contribute to RA pathology by either activation of macrophages, activating osteoclasts by immune complex formation, or directly promoting bone loss via binding to citrullinated vimentin found in bone membranes [1,103].

Studies showing a correlation between the presence of ACPAs and the risk of developing bone erosions have suggested ACPAs to be involved in bone erosion [111,112,113]. Here, ACPAs may increase bone resorption by either (1) immune complex-mediated activation of macrophages which in turn secrete pro-inflammatory cytokines (e.g., TNF-α, RANK-L) promoting the differentiation of osteoclasts (see above) or (2) the direct recognition of citrullinated proteins on the surface of osteoclast precursor cells resulting in efficient osteoclast generation (reviewed in [114]). Moreover, both IL-8-dependent osteoclast differentiation and activation by ACPAs was related to joint pain [115], suggesting that ACPAs also actively contribute to the development of joint pain.

However, not all ACPAs seem to be equally detrimental in RA, since ACPAs are usually present in patient´s sera for years without causing disease symptoms. Here, recent data suggested that differences in ACPA glycosylation patterns might account for the observed differences between pathologic and non-pathologic ACPAs [114,116]. Especially hyposialysation at position Asn297 in the fragment crystallizable region of ACPAs was shown to generate highly pathogenic ACPAs [116], and endoglycosylase treatment of IgG antibodies was shown to reduce the severity of collagen type II-induced RA in a mouse model [117]. Since the constant region of an antibody is mainly responsible for the respective antibody’s effector function, differences in glycosylation pattern can significantly change the antibody´s biological effects even when the variable, antigen-binding part of the respective antibody is unchanged. 

In line with these results, both galactosylation and sialylation levels of ACPAs were shown to decrease in ACPA-positive RA patients in the timeframe shortly before first onset of RA symptoms [118,119,120]. Mechanistically, IL-21 and IL-22 produced by Th17 cells in the inflamed joint were suggested to trigger the release of pathogenic, hyposialysated ACPAs by reducing expression of the enzyme sialyltransferase ST6GAL1 [114].

### 3.5. RA also Results in Neovascularization

Moreover, the inflammatory processes in the joints of RA patients are often paralleled by neovascularization (growth of new blood vessels) and synoviocyte hyperplasia (excessive proliferation of synoviocytes) [121,122].

The prolonged exposure of synoviocytes to the inflammatory milieu of the arthritic joint was shown to result in a transformed, cancer-like phenotype characterized by both uncontrolled proliferation and reduced sensitivity towards apoptosis [123,124]. This phenotype was suggested to result from an accumulation of mutations and epigenetic changes, e.g., somatic mutation of the tumor suppressor gene p53 in RA synovium rather promoting p21-mediated cell cycle arrest than apoptosis [125,126,127,128] under these inflammatory conditions. 

In light of the observation, that later stages of RA may be refractory to immunological treatment approaches, these results suggest that late stage RA may have characteristics of a cell-autonomous genetic and epigenetic synoviocyte disease, characterized by altered cell death pathways [128].

## 4. Diagnosis of RA

Clinically, RA patients typically present with a recent onset of tender and swollen joints, morning joint stiffness, generalized sickness symptoms (see above), as well as abnormal laboratory tests [1]. Timely and precise diagnosis is of high importance in RA treatment, since early diagnosis can arrest disease in many patients, thereby preventing or substantially slowing disease progression, irreparable joint damage, and disability in up to 90% of RA patients [7].

Typically, RA is diagnosed by a combination of patient’s symptoms, results of doctor´s examination, assessment of risk factors, family history, joint assessment by ultrasound sonography, and assessment of laboratory markers such as elevated levels of CRP and ESR in serum and detection of RA-specific autoantibodies (already discussed above) [7,129].

Both ultrasound and MRI have been recommended for diagnosing and monitoring disease activity in RA patients [130]. Ultrasound analysis (e.g., as high-resolution musculoskeletal ultrasound) of inflamed joints allows imaging of synovial proliferation by grayscale as well as both active inflammation and neoangiogenesis by power Doppler [131]. In addition, ultrasound is able to identify bone erosions [132], as well as subclinical synovitis that may result in radiographic disease progression even if the patient is in apparent clinical remission [133,134]. Due to these capabilities, ultrasound is widely used in clinical practice as well as in clinical trials for the diagnosis of RA and the monitoring of disease states [135]. 

The advantages of ultrasound are its relatively low cost, wide availability, lack of contraindications, and non-invasive real-time imaging capabilities. Disadvantages are that ultrasound is considered an operator-dependent technology because of it being training-intensive in terms of both measurement and quality assessment [135].

While being a very sensitive diagnostic tool to detect e.g., synovial hypertrophy or pannus formation before the occurrence of bone erosion, routine usage of magnetic resonance imaging (MRI) techniques (preferably contrasted) in the diagnosis of RA is limited by cost factors and the limited capacity to image multiple joints in one measurement [2,136].

As clinical biomarkers, CRP and ESR are routinely used to determine the general inflammatory state of RA patients. CRP is an acute phase reactant, composed of five 23-kDa subunits belonging to the pentraxin protein family. Its serum concentration can increase by three or more log steps in the presence of either infection, inflammation, or tissue injury [137,138]. Triggered by the cytokines IL-6, IL-1β, and TNF-α, the main sources of CRP production are hepatocytes, but also to a lower extent vascular smooth muscle cells, monocytes, lymphocytes, adipocytes, and neurons [138,139,140,141,142].

As immunological effector function, CRP both activates the classical complement pathway and stimulates the influx and phagocytotic activity of neutrophils [143,144]. Moreover, interaction of CRP with the Fc gamma receptors FcγRI and FcγRIIA was shown to promote both survival and proliferation of macrophages as well as pro-inflammatory cytokine production and subsequent amplification of inflammation via production of MMPs, secretion of monocyte chemoattractant protein-1 (MCP-1) and macrophage colony-stimulating factor (M-CSF), and inhibition of the anti-inflammatory cytokine IL-10 [145,146,147,148,149,150]. Moreover, RANK-L expression by PBMCs induced by CRP also resulted in the differentiation of osteoclast precursors into osteoclasts [151].

While being unaffected by factors like age, gender, and abnormalities in erythrocytes and serum proteins [151], CRP levels were found to positively correlate with both disease activity, histological changes in the synovium, and radiological progression and clinical parameters such as morning stiffness, pain, fatigue, grip strength, articular index, and disability [152,153,154,155]. Therefore, CRP was found to be a useful marker in RA diagnosis, as well as the monitoring of disease progression and prognosis of joint damage [156,157,158].

ESR is a widely used standard laboratory test determining the speed at which erythrocytes settle within a test tube containing a blood sample of the respective patient [159]. In the presence of inflammatory processes, infections, autoimmune disorders (e.g., RA), but also pregnancy, anemia, certain kidney diseases, and some cancers (e.g., lymphoma and multiple myeloma) increased concentrations of fibrinogen in the blood cause a coagulation of the red blood cells (reviewed in [160]). In this process, the erythrocytes form stacks called “rouleaux”, which settle faster in the test tube because of their increased density [160].

### EULAR Criteria for the Diagnosis of RA

Although primarily developed for the identification of homogenous patient populations in clinical RA trials [17,161], the 2010 ACR-EULAR (American College of Rheumatology-European League against Rheumatism) criteria can also be used by physicians to diagnose RA [7,17,161]. Hereby, the 2010 EULAR criteria incorporate all of the above discussed diagnostic parameters: joint involvement, abnormalities in CRP and ESR, presence of RA-specific autoantibodies, and overall symptom duration.

**Figure 3:**
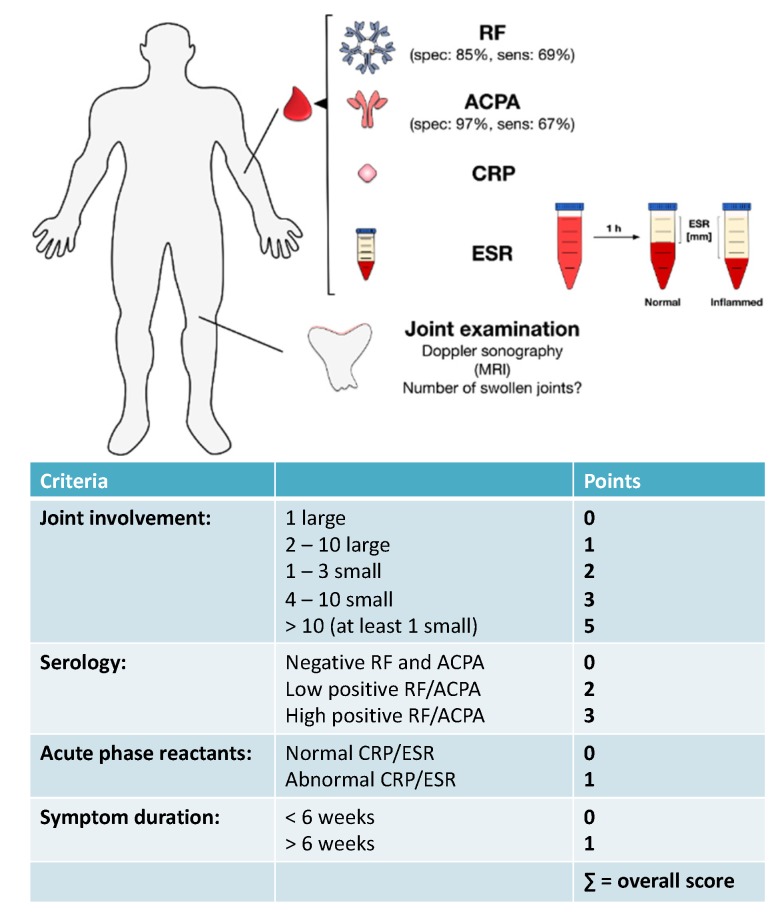
Clinical parameters frequently used in the diagnosis of RA and their quantification using the 2010 ACR-EULAR (American College of Rheumatology-European League against Rheumatism) classification criteria. Clinical diagnosis (left) of RA relies on joint examination (mainly via sonography, but also by magnetic resonance imaging (MRI)), and the serological determination of RA-specific autoantibodies (Rehmatoid factor (RF) and ACPAs) and detection of elevated levels of C-reactive protein (CRP) and an increased erythrocyte sedimentation rate (ESR). The 2010 ACR-EULAR RA classification criteria (right). Scoring parameters are number and size of the involved joints, the presence and concentration of RA-specific ACPAs and RF autoantibodies, presence of abnormal levels of CRP and increased ESR, and overall duration of disease symptoms. According to the 2010 ACR-EULAR RA classification criteria a RA diagnosis is made if the overall score is greater than six and other causes for synovitis (see above) can be excluded.

Using the 2010 EULAR criteria joint involvement is graded with 0–5 points depending on the number and size of the involved joints (requiring the presence of at least one clinically swollen joint), up to three points are given depending on the presence and concentration of ACPAs and RF autoantibodies, and one point each for presence of abnormal levels of CRP and increased ESR as well as the overall duration of disease symptoms (Figure 3). This classification allows for a maximum disease score of 10 points (see Figure 3) and a RA diagnosis is made if (1) the overall score of the patient is greater than six and (2) other causes for synovitis (e.g., other inflammatory arthritic conditions, infection, or trauma) can be excluded [2]. Overall, the sensitivity of the 2010 EULAR criteria was reported to be 82% with a specificity of 61% [17].

## 5. Treatment of RA

Once RA is diagnosed in a patient, the overall treatment target is to either reach full remission or at least significantly lower disease activity within a span of approximately 6 months in order to prevent joint damage, disability, and systemic manifestations of RA [7,129].

The importance of prompt and targeted RA treatment is underlined by the fact that 80% of insufficiently treated patients will have misaligned joints and 40% of patients will be unable to work within 10 years of disease onset [7,162,163].

To achieve the treatment goals, treatment should be initiated promptly and continuously with frequent reassessment of both the state of the disease and the effectiveness of the applied treatment strategy. Until the early 1990s the common treatment strategy of RA was based on a treatment pyramid consisting of bed rest, the administration of non-steroidal anti-inflammatory drugs (NSAIDs), and if these treatments failed disease-modifying anti-rheumatic drug (DMARD) therapy [129]. However, the efficacy of this treatment strategy was limited and within years rheumatoid arthritis frequently resulted in joint destruction, disability, inability to work, and increased mortality [164].

**Figure 4:**
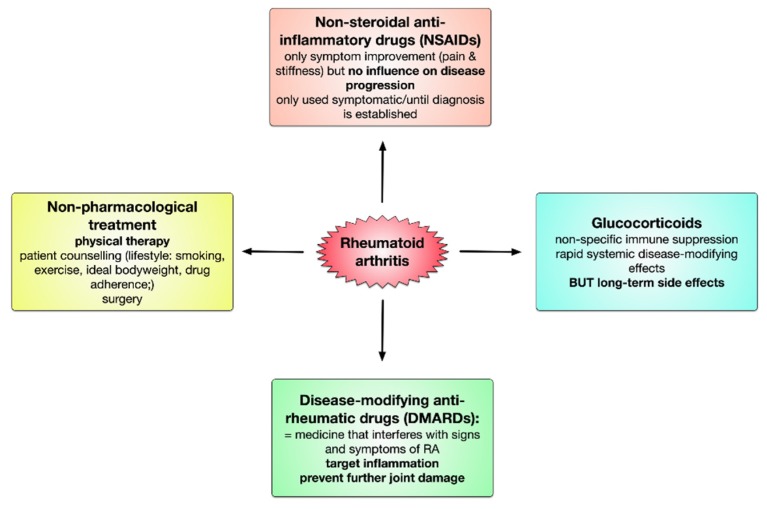
Overview over the available treatment strategies for RA patients. The possible treatments for RA are divided into four main strategies. Non-pharmacological treatments include a combination of physical therapy, patient counseling in lifestyle factors, and surgical procedures to remove and/or replace the affected joint and bone areas. Non-steroidal anti-inflammatory drugs (NSAIDs) are usually used only for symptomatic treatment and/or until the RA diagnosis is established since these drugs reduce pain and stiffness in the affected patients but have no influence on disease progression. In contrast to this, non-specific immune system suppression via the application of glucocorticoids has rapid disease-modifying effects but its long-term usage is limited due to severe side-effects. Finally, disease-modifying anti-rheumatic drugs (DMARDs) are used to target inflammation and prevent further joint damage and disease progression.

Fortunately, the repertoire of therapeutic drugs with benefit in the treatment of RA has grown steadily in the last 30 years. Currently, the available drug classes include NSAIDs, immunosuppressive glucocorticoids, and DMARDs. Drug treatment is typically supplemented by non-pharmacological treatment which includes physical therapy to sustain joint mobility and patient counselling to slow down disease progression (see Figure 4).

NSAIDs like for example aspirin, diclofenac, or ibuprofen effectively reduce pain and swelling and improve joint function but are not disease-modifying since they do not prevent additional joint damage [1]. Mechanistically, the anti-inflammatory properties of NSAIDs can be mainly attributed to the inhibition of prostanoid biosynthesis [165]. Prostanoids, such as for example prostaglandin (PG) E2, PGD2, PGF2α, thromboxane A2, and prostacyclin, are second messengers that interact with and activate surface expressed G-protein coupled receptors thereby modulating many cellular functions. While effectively reducing RA symptoms, the application of NSAIDs is frequently accompanied by renal-, hepatic-, gastrointestinal-, and cardiovascular side-effects (reviewed in [166]).

Glucocorticoids like prednisolone are highly potent anti-inflammatory drugs that delay radiologic progression in early disease stages by general suppression of gene expression [2,167]. Despite these beneficial effects, the disease-modifying effects of glucocorticoids were described to be minimal and the long-term application of glucocorticoids is hampered by severe multisystemic metabolic side-effects such as gastrointestinal bleeding, osteoporosis, and ulcer formation [1,2,168].

Finally, DMARDs are drugs that target rheumatoid inflammation and thereby prevent further joint damage. Per definition DMARDs are drugs that, in contrast to drugs which do not prevent disease progression (e.g., NSAIDs or pain medication), interfere with the signs and symptoms of RA, improve physical function, and inhibit progression of structural joint damage [1,7].

**Figure 5:**
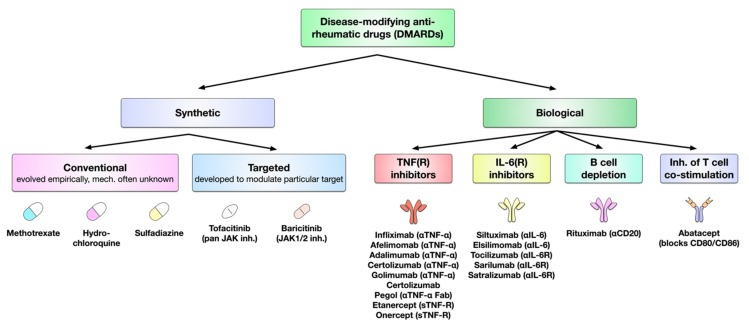
Overview over the currently available DMARDs. The different DMARDs are grouped into synthetic (further subdivided into conventional and targeted synthetic DMARDs) and biological DMARDs. Biological, antibody-based, DMARDs include anti-TNF-α and anti-TNF-R antibodies, anti-IL-6- and anti-IL-6R antibodies, B cell depleting anti-CD20 antibodies, as well as inhibitors of T cell co-stimulation. Abbreviations: JAK: Janus activated kinase, αTNF-α/αIL-6: anti-TNF-α/IL-6 antibody, sTNF-R: anti soluble TNF receptor antibody, αIL-6R: anti Il-6 receptor antibody, Fab: antibody fragment, inh.: inhibitor.

The available DMARDs are further subdivided into (1) conventional synthetic DMARDs (methotrexate, hydrochloroquine, and sulfadiazine), (2) targeted synthetic DMARDs (pan-JAK- and JAK1/2-inhibitors), and (3) biologic DMARDs (TNF-α inhibitors, TNF-receptor ® inhibitors, IL-6 inhibitors, IL-6R inhibitors, B cell depleting antibodies, and inhibitors of co-stimulatory molecules) (Figure 5). The following sections will discuss the different available DMARDs in more detail.

## 6. DMARDs in the Treatment of RA

Box 1 **Commonly used scores to quantify effectiveness of RA treatment.**

**American College of Rheumatology response criteria 20/50/70 (ACR20/50/70):** Composite measure defined as either 20%/50%/70% improvement in both number of tender and swollen joints combined with 20%/50%/70% improvement in three of the following five criteria: (1) patient global assessment, (2) physician global assessment, (3) functional ability measure (via patient questionnaire), (4) visual analog pain scale, and (5) ESR/CRP level.

**Disease Activity Score 28 (DAS28):** Composite score including four disease parameters: (1) number of swollen joints (maximum is 28), (2) number of tender joints (maximum is 28), (3) increased ESR, and (4) patient global assessment. The overall score is calculated by a complex mathematical formula and scores greater than 5.1 suggest active disease, less than 3.2 low disease activity, and less than 2.6 indicate a state of remission.

**Disease Activity Score 28-C-Reactive Protein (DAS28-CRP)**: DAS28 score that uses CRP as general inflammation parameter instead of ESR.

### 6.1. Conventional Synthetic DMARDs

Currently, the class of synthetic DMARDs mainly comprises the three most frequently used substances methotrexate, hydrochloroquine, and sulfadiazine. Developed empirically, the definitive mode of action of these three drugs is still unknown.

#### 6.1.1. Methotrexate

After RA diagnosis, methotrexate is the suggested first-line therapy for RA. Accordingly, for more than 20 years methotrexate has been predominantly used for the therapy for RA in the United States [169,170]. Initially, it is suggested to be co-applied with low doses of glucocorticoids to efficiently and timely reduce the levels of joint inflammation [7]. With this initial treatment regimen 30%–50% of early RA patients are able to reach a state of either remission or low disease activity [7,171,172,173]. In addition to its high effectiveness, the safety and toxicity profile of methotrexate is well known, and the costs of treatment are low if for example compared to targeted synthetic or biological DMARD therapy [174]. Moreover, the efficacy of both targeted synthetic of biological DMARDs is increased if these drugs are applied in combination with methotrexate, making methotrexate a staple in long-term RA treatment [7,175].

Although its definitive mode of action is currently still unknown, several effector mechanisms were suggested to contribute to the anti-inflammatory properties of methotrexate: structurally, methotrexate is an analogue of folic acid that interferes with the activity of the dihydrofolate reductase, thereby both inhibiting nucleotide synthesis and purine metabolism [176,177,178]. By these actions, it is resulting in the production and release of adenosine, which was shown to have direct anti-inflammatory properties [2,176,177,178].

Of note, many of the side-effects of methotrexate such as hair loss, stomatitis, nausea, and its hepatotoxicity are directly caused by its disruption of folate metabolism and can be prevented by the prophylactic supplementation of folate (mainly as folic acid) in patients treated with methotrexate [7,179].

Methotrexate was speculated to decrease tissue damage in RA patients by either suppressing the accumulation of toxic compounds via tetrahydrofolate [169,170,180] or by decreasing glutathione-mediated tissue damage caused by toxic oxygen metabolites [170,181].

Additionally, methotrexate was reported to inhibit the binding of IL-1ß to the IL-1ßR, preventing IL-1ß-induced inflammatory responses [182]. Moreover, methotrexate was suggested to have effects on many other enzymes such as for example methyltransferases (which are important in both B- and T cell function) [183].

#### 6.1.2. Sulfasalazin

First produced in Sweden in the 1930s, Sulfasalazine was introduced in RA therapy because of its antibiotic activity and the supposed contribution of bacterial/viral infection to RA establishment [170]. Sulfasalazin, which can be efficacious in the treatment of moderate RA [2,184], is a pro-drug that is metabolized in vivo by intestinal bacteria into its two active components sulfapyridine and 5-aminosalicylic acid [185].

While their exact mechanism of action is unknown, both sulfapyridine and 5-aminosalicylic acid were shown to have anti-inflammatory, immune-modulatory, and antibiotic properties [186,187]. However, sulfapyridine was suggested to be the major therapeutic component of sulfasalazine [186,187].

Clinically, sulfasalazine was shown to significantly improve RA treatment in comparison to placebo, improving articular index (46% improvement with sulfasalazine compared to 20% for placebo) while reducing morning stiffness (61% improvement with sulfasalazine compared to 33% for placebo), number of swollen joints (59% reduction for sulfasalazine vs. 33% for placebo), pain (42% for sulfasalazine vs. 15% for placebo), as well as patient global self-assessment (26% for sulfasalazine vs. 1% for placebo) [188,189,190]. Compared to hydrochloroquine treatment, sulfasalazine tended to improve number of swollen joints, pain, ESR (43% reduction for sulfasalazine vs. 26% for hydrochloroquine), and duration of morning stiffness (59% reduction for sulfasalazine vs. 40% for hydrochloroquine), while resulting in lower drop-out numbers due to lack of drug efficacy (5% for sulfasalazine vs. 15% for hydrochloroquine) [189,190].

Typical side-effects of sulfasalazine include fatigue, CNS reactions, nausea, abdominal pain (dyspepsia), diarrhea, hypersensitivity reactions, and with a lower frequency neutropenia, thrombocytopenia, and pan-hypogammaglobulinaemia [191,192,193,194].

#### 6.1.3. Hydrochloroquine

Chloroquine and hydroxychloroquine, which are primarily used as antimalarial drugs, also display anti-inflammatory and immunomodulatory properties which make these substances suitable for the treatment of mild cases of inflammatory arthritic diseases. Of note, in the treatment of RA, hydrochloroquine was shown to only have limited structural effects on joint damage [1,195].

The anti-inflammatory properties of the lipophilic hydroxychloroquine are likely mediated by its inhibition of both lysosomal antigen degradation and subsequent reduction in the surface presentation of antigen-derived peptide:MHC II complexes on APCs [196]. Together, these effects of hydrochloroquine can prevent the activation of autoreactive T cells and subsequent inflammatory responses. Furthermore, hydroxychloroquine also inhibits the production of RF antibodies and acute phase reactants as well as many different enzymes including collagenase and proteases (which directly cause cartilage breakdown) (reviewed in [197]).

Meta-analysis of four pooled clinical trials including approx. 600 total patients by Suarez-Almanor et al. showed that hydrochloroquine treatment provided a significant benefit in RA patients in comparison to placebo treatment without increasing the frequency of withdrawals due to either lack of efficacy or toxicity [198]. In these studies, hydrochloroquine was shown to improve the following outcome measures: tender joints, swollen joints, pain, both physician and patient global assessment (differences in standardized mean differences compared to placebo: tender joints: −0.33, swollen joints: −0.52, pain: −0.45, physician global assessment; −0.45, patient global assessment: −0.39), as well as ESR (weighted mean difference of 6 mm compared to placebo treatment) [198].

Ophthalmic toxicity is the most important side-effect in RA patients after treatment with either chloroquine or hydroxychloroquine (frequency: 4.4%–19%) [199,200]. Importantly, retinal degeneration was shown to gradually progress even after cessation of chloroquine therapy [199,201].

#### 6.1.4. Triple Therapy with Synthetic DMARDs

Importantly, synthetic DMARDs can be co-applied simultaneously. Indeed, triple therapy with methotrexate, sulfasalazine, and hydroxychloroquine was a mainstay of RA therapy before biological and targeted synthetic DMARDs were available [1,202]. Here, even triple therapy is more cost effective than the application of biological DMARDs (see below for costs of biological DMARDs) and recently triple therapy was shown to be as efficient as the combination treatment with methotrexate and the TNF-blocker etanercept (mean DAS scores 3.8 ± 1.4 for triple therapy vs. 3.5 ± 1.3 methotrexate plus etanercept) in patients that did not respond to monotherapy with methotrexate [1,202]. Moreover, meta-analyses by Graudal et al. showed, that in preventing structural joint damage, combination treatment with two to three conventional synthetic DMARDs was not inferior to treatment with the combination of one biological DMARD plus one synthetic DMARD [203]. Here, no differences in reduction of ACR response (see Box 1), disease progression, disability, and withdrawals due to lack of efficacy were observed [203]. In line with this, several studies demonstrated comparable treatment efficacies when comparing triple therapy with the combination of a TNF-α inhibitor plus methotrexate [204,205,206,207].

However, tolerability and drug-induced liver toxicity are factors limiting the utility of triple therapy. Liver toxicity with either chronically elevated levels of aminotransferases or hepatic fatty infiltration with fibrosis that can progress to liver cirrhosis are known complications in long-term treatment with methotrexate [208,209]. Sulfasalazine also causes acute clinically relevant liver damage (jaundice, hepatic failure) in one in 1000 patients [210]. Cummings et al. assessed the tolerability, longevity, and efficacy of triple therapy in 119 early-onset RA patients [211]. In this study retention on standard triple therapy was reported to be 39 weeks [211]. Of the 119 patients starting triple therapy only 32% remained on triple therapy at last follow-up (median duration of treatment: 70 weeks). Reported reasons for first DMARDs withdrawal were adverse event (38%), active disease requiring switching of drugs (28%), remission (7%), and non-adherence (4%) [211]. Among adverse events, sulfosalazin was reported to be the most frequent cause for drug withdrawal (49%), followed by methotrexate (29%) and hydrochloroquine (13%) while the most common adverse events were upper gastrointestinal intolerance (14%) and either allergy or rash (11%) [211].

### 6.2. Targeted Synthetic DMARDs

In contrast to the aforementioned conventional synthetic DMARDs, targeted synthetic DMARDs were developed specifically to target a key step in the cytokine-mediated induction of inflammatory responses, namely the JAK-STAT pathway.

**Figure 6:**
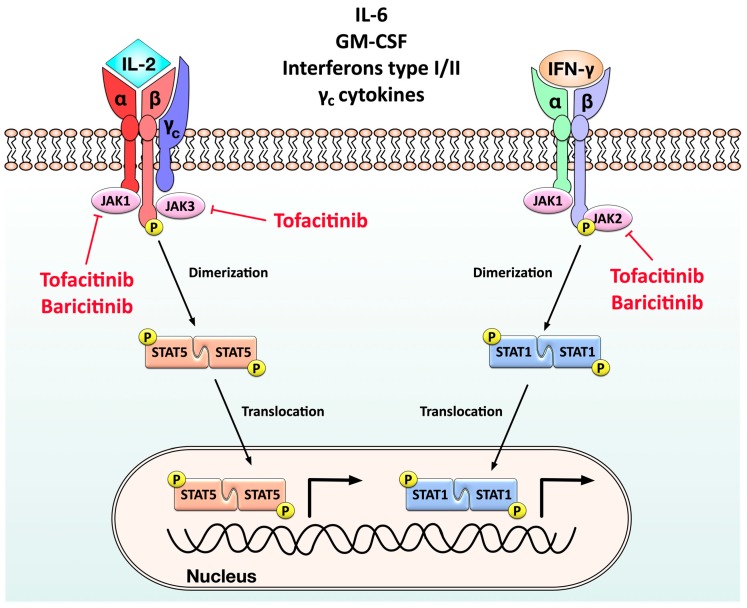
Molecular mode of action of Janus activated kinase (JAK) inhibitors. Binding of pro-inflammatory cytokines (e.g., IL-6, common γ-chain-containing cytokines (e.g., IL-2 or IL-15), type I and II interferons, or granulocyte-monocyte colony stimulating factor (GM-CSF)) to their respective receptors on the surface of immune cells triggers the recruitment of JAKs. JAKs subsequently phosphorylate the intracytoplasmic parts of the respective receptors, inducing the phosphorylation, auto-homodimerization, and nuclear translocation of different signal transducer and activator of transcription (STAT) molecules. In the respective cell’s nucleus STAT dimers promote the expression of many pro-inflammatory genes that initiate and sustain joint inflammation and tissue damage. The targeted DMARDs tofacitinib and baricitinib inhibit this activation of JAKs and thereby prevent immune cell activation and subsequent inflammatory responses.

Binding of either pro-inflammatory cytokines (e.g., IL-6, common γ-chain containing cytokines like IL-2 or IL-15, type I and II interferons, or granulocyte-monocyte colony stimulating factor (GM-CSF)) to their respective receptors on the surface of immune cells triggers both the recruitment of JAKs to the respective cytokine receptors and phosphorylation of the intracytoplasmic parts of these receptors by the JAKs (reviewed in [212]) (Figure 6). This phosphorylation in turn subsequently induces the phosphorylation of different STATs (Figure 6). The phosphorylated STATs then undergo a homodimerization which triggers their translocation in the respective cell’s nucleus where they promote the expression of many pro-inflammatory genes that can initiate and sustain both joint inflammation and tissue damage (reviewed in [212]) (Figure 6).

Over the last years, drugs that inhibit the different JAKs have been a significant improvement in the treatment of RA [129]. Tofacitinib, the first JAK inhibitor that was approved in many countries, is a pan-JAK inhibitor [129] that prevents the recruitment and activation of JAK1, JAK2, and JAK3 (although with higher inhibitory activity towards JAK1/2 than JAK3) and therefore the downstream activation of STAT1 and STAT5 [213]. In contrast to this, baricitinib is a specific JAK1/2-inhibitor [213].

Since all JAK inhibitors basically target different molecules belonging to the same pathway, it is readily understandable that all of these drugs have similar treatment efficacies and side-effects in RA patients [214].

In a 52-week, phase 3, double-blind, placebo- and active-controlled trial including 1307 patients with active RA that were receiving background therapy with methotrexate, 70% of patients treated with 4 mg baricitinib daily (a dose twice as high as the 2 mg dose approved by the FDA) reached ACR20 at week 12 compared to 40% with placebo [215]. Baricitinib also inhibited radiographic progression of joint damage (mean change of modified total Sharp score from baseline, 0.41 for baricitinib vs. 0.90 for placebo) [215]. While randomized, placebo-controlled trials have proven JAK inhibitors to be effective with an acceptable safety profile, it is noteworthy that the application of JAK inhibitors is frequently accompanied by side-effects such as increased frequency of infections (often with Herpes zoster), formation of blood clots, elevation of blood cholesterol levels, cytopenia, and gastrointestinal side-effects (bowel perforation) [214,216].

Clinically, tofacitinib and baricitinib can both be used as monotherapy or co-applied simultaneously with methotrexate depending on the individual patient’s response to treatment [129].

### 6.3. Biologic DMARDs

The currently approved biological DMARDs have four underlying modes of action: (1) the neutralization of either TNF-α or the TNF receptor, (2) the neutralization of IL-6 directly or the blockage of the IL-6R and the associated inflammatory signaling, (3) the inhibition of T cell co-stimulation by APCs, and (4) the depletion of B cells [217].

#### 6.3.1. TNF-α Inhibitors

TNF-α neutralizing drugs are subdivided into neutralizing monoclonal antibodies (afelimomab, infliximab, certolizumab, adalimumab, golimumab), antibody fragments (certolizumab pegol), and soluble TNF receptor constructs (etanercept, onercept) [1]. TNF-α inhibitors were approved by the US Food and Drug Administration for RA therapy in the following order: etanercept (1998), infliximab (1999), adalimumab (2002), certolizumab pegol (2009), and golimumab (2009).

By neutralizing TNF-α and the inflammatory processes induced by this cytokine, these substances effectively suppress joint inflammation as well as both cartilage and bone damage. TNF-α inhibitors can be used in combination with methotrexate or other DMARDs, and are also frequently used as second-line treatments when patients fail to respond to synthetic DMARDs monotherapy.

Clinically, the TNF-α neutralizing antibody infliximab (a chimeric mouse-anti human IgG1) was shown to downregulate the production of other pro-inflammatory cytokines (IL-6 for days 1–28 compared to placebo control), reduce both leucocyte trafficking and tissue destruction, and to lead to both hematological normalization and normalized T cell responses [218]. However, this clinical efficacy is paralleled by frequently observed side-effects such as increased frequencies of infections [207,219,220] and non-melanoma skin cancers [221,222,223,224,225], but not other types of cancer [223,226,227,228,229].

Adalimumab is a human IgG1 monoclonal antibody that binds both soluble and transmembrane TNF-α. Several clinical trials have shown that treatment of RA patients with adalimumab results in better ACR20 responses, reduction of swollen and tender joint counts, and a decrease in mean CRP levels [230,231,232,233]. Here, adalimumab improved ACR20 (52.8% for adalimumab vs. 34.9% for placebo), ACR50 (28.9% vs. 11.3%), and ACR70 (14.8% vs. 3.5%) responses at week 24 compared with the placebo-treated group [230]. Adalimumab administration also reduced numbers of swollen and tender joint counts, and decreased mean CRP levels at week 52 while CRP concentrations remained elevated at twice the normal range in placebo-controls [233]. A 10-year clinical trial also showed that patients with baseline disease duration ≤2 years who were treated with adalimumab, had both better ACR50 responses (71.9% of adalimumab treated patients vs. 52.9% in the placebo control group) and physical function (Health Assessment Questionnaire without Disability Index (HAQ-DI) <0.5 in 60.6% of adalimumab treated patients vs. 39.5% in the placebo control), highlighting the benefit of early treatment [234]. As one of the second-line choices for RA treatment, patients who have failed synthetic DMARD therapy often show significant and rapid improvement of several RA-related disease activities with adalimumab monotherapy [231]. Here, patients treated weekly with 40 mg of adalimumab had higher ACR50 (35.0% for adalimumab vs. 8.2% for placebo) and EULAR response rates (63.1% vs. 26.4%) as well as lower mean HAQ-DI scores (−0.49 vs. −0.07) compared to placebo controls [231].

A clinical analysis of 14,109 patients aggregated from 71 individual adalimumab clinical trials showed that the most frequently reported serious adverse events were infections (incidence rates: 4.6/100 patient-years), pneumonia (0.7/100 patient-years), and cellulitis (0.3/100 patient-years) [235].

Different from infliximab and adalimumab, which are both full-length IgG1 antibodies, certolizumab pegol only consist of a Fab fragment recognizing TNF-α, conjugated with polyethylene glycol (PEG) to extend its half-life. A clinical study reported that patients previously not responding to synthetic DMARD therapy, showed improved ACR50 and ACR70 responses compared to placebo at week 24 (ACR50: 22.7% for certolizumab pegol vs. 3.7% in the placebo group, ACR70: 5.5% vs. 0%) [231]. In addition, physical function, arthritis pain, and fatigue were also improved after treatment for 24 weeks [231].

No significant life-threatening side-effects were reported from certolizumab pegol-treated RA patients. The reported side-effects included fatigue, mild skin rash, or mild upper respiratory tract infections [236].

Etanercept is a fusion protein combining a TNF-α receptor-2 p75 subunit with the Fc domain of a human IgG1 molecule which mediates the formation of TNF-α R2 p75:huIgG1 Fc homodimers. A clinical study involving 180 patients revealed a dose–response effect on both swollen and tender joints (number of swollen joints for placebo/0.25/2/16 mg etanercept treatment groups, 22/24/17/13) [237]. Moreover, treatment with etanercept improved pain and reduced duration of morning stiffness in RA patients (pain evaluation and hours of morning stiffness for placebo/0.25/2/16 mg etanercept treatment groups: 6.1/5.6/4.6/3.1 and 4.1/5.3/2.6/1.1, respectively) [237]. 

During clinical trials etanercept was well tolerated by RA patients. The percentage of treatment discontinuations due to adverse effects was reported with approximately 4% [238]. Here, the most frequently reported side-effects were non-upper respiratory tract infections (38%), injection site reactions (37%), upper respiratory tract infections (29%), headache (17%), and rhinitis (12%) [238].

There are several clinical studies comparing treatment efficacy and side-effects between infliximab, adalimumab, and etanercept [239,240,241,242]. Here, it was shown that TNF-α neutralizing antibodies possess a high potential to induce the production of anti-drug antibodies (ADAs) (detection of ADAs within 18 months of treatment in either the adalimumab (19.2%–31.2% of patients) or infliximab group (17.4–29.4% of patients)) [239,240,241,243]. ADAs can not only decrease drug levels in serum, but also raise safety concerns like induction of type I–III hypersensitivity responses [244]. A clinical study also showed that presence of anti-adalimumab ADAs increased the risk of developing thromboembolism [245].

The lack of the human Fc domain in certolizumab pegol reduces the possibility for ADA generation. Consequently, clinical results demonstrated that none or only low levels of ADAs were detected in certolizumab pegol-treated patients [241], while several studies indicated a high immunogenicity of both infliximab and adalimumab which might lead to the development of ADAs [239,240,241,243]. The induction of ADAs against adalimumab and infliximab is associated with both major and minor clinical adverse effects [243]. Moreover, the production of ADAs is also correlated with some disease outcomes, such as the inflammation markers ESR (*p* = 0.0080) and CRP (*p* = 0.0011), which were shown to be significantly different between patients with and without the presence of ADAs [240].

Although there are side-effects or reports of ADA formation, taken together, all of the clinical studies still suggest, that anti-TNF-α neutralizing drugs have the capacity to significantly improve disease symptoms in RA patients compared to placebo treatment.

#### 6.3.2. IL-6 Inhibitors, IL-6R Inhibitors

The development of IL-6 blockers provides another possibility for RA treatment. Monoclonal antibodies currently used in RA patients to inhibit IL-6 signaling are subdivided into (1) antibodies directly neutralizing IL-6 (elsilimomab, siltuximab, sirukumab) and (2) antibodies binding to the IL-6R blocking the pro-inflammatory signaling induced by IL-6 binding (tocilizumab, satralizumab, sarilumab).

Pro-inflammatory signaling induced by IL-6 is mediated via the binding of IL-6 to the soluble IL-6 receptor (sIL-6R) which subsequently forms a trimer with two transmembrane glycoprotein (gp) 130 subunits [134]. This complex of IL-6, sIL-6R, and two molecules of gp130 in turn mediates JAK activation and subsequent phosphorylation, homodimerization, and nuclear translocation of STAT-3 driving pro-inflammatory gene expression [135].

Tocilizumab is a humanized monoclonal antibody binding to the human IL-6R and therefore inhibiting IL-6 signaling [246]. Besides sarilumab (also binding to the IL-6R), it is the only approved anti-IL-6(R) antibody for the treatment of RA [247]. Both, tocilizumab and sarilumab are widely used in the treatment of RA [247]. Potential immunological effects of tocilizumab on RA include: (1) induction and expansion of B-regulatory cells, (2) reduction of pro-inflammatory cytokines, (3) decrease of T cell-related cytokine secretion as well as IL-21 production from memory/activated CD4^+^ cells, (4) downregulation of chemokine genes, (5) induction of genes associated with synovial fluid healing, and (6) increasing osteoprotegerin expression (likely blocking RANK-L-RANK signaling and inhibiting bone resorption) [248,249]. Interestingly, during tocilizumab treatment, serum concentrations of both IL-6 (58.4 ± 13.8 pg/mL at baseline vs. 92.8 ± 82.4 pg/mL at day 14) and sIL-6R (27.7 ± 4.4 ng/mL at baseline vs. 251.4 ± 24.7 ng/mL at day 42) were shown to significantly increase [250].

Clinical research suggests that tocilizumab does not inhibit IL-6 production directly, instead, as long as free tocilizumab is detectable, the sIL-6R is saturated with tocilizumab [250]. This tocilizumab-sIL-6R immune complex in turn extends the half-life of sIL-6R and inhibits sIL-6R-mediated catabolism of IL-6, resulting in increased serum concentrations of both IL-6 and sIL-6R [250]. Clinically, tocilizumab shows beneficial effects in many RA patients, including patients with an insufficient response to traditional synthetic DMARDs, methotrexate, or TNF-α inhibitors [251]. These effects include improvement of RA symptoms, reduction of ESR (−3.3 mm compared to baseline before treatment) and mean CRP levels (−10.27 in tocilizumab treated patients vs. −3.0 in the group with continuous TNF-inhibitor treatment), reduced arterial stiffness, and higher ACR20/50/70 (47.3%/20.9%/8.1% of patients reaching criteria) response rates [248,249].

The most common side-effect of tocilizumab application are skin- and subcutaneous infections [251]. Nevertheless, infection rates are rather low and comparable to those observed upon treatment with anti-TNF-α antibodies [251]. Other adverse effects include dyslipidemia, neutropenia, thrombocytopenia, and enhanced levels of liver enzymes [252]. While tocilizumab’s overall efficacy and safety profiles are similar to TNF-α blockers when combined with other DMARDs (such as methotrexate), tocilizumab also shows differences especially in its potential when used as monotherapy, such as low production of ADAs and more effective improvement of certain disease symptoms such as anemia and fatigue [252].

Sarilumab is a fully humanized monoclonal antibody that also binds to IL-6R. Compared with tocilizumab, sarilumab has both a 15–22-fold higher binding affinity to IL-6R and a prolonged half-life [253]. However, the overall efficacy and safety of sarilumab appears to be comparable with tocilizumab, with for example mean CRP levels (23.8 for sarilumab vs. 24.9 for tocilizumab), tender joint counts (24.7 vs. 23.5), HAQ-DI (1.71 vs. 1.78), and incidence of reported adverse effect (70.6% vs. 66.7%) being nearly identical between both drugs [254,255]. In addition, preclinical trials demonstrated, that treatment with sarilumab resulted in reduced loss of cartilage matrix components, as well as reductions in both inflammatory- (synovitis and pannus formation) and erosive (bone erosion and loss of tissue architecture) parameters compared to control antibody treatment [256].

Infections, elevations in alanine aminotransferase levels, and neutropenia were the most common side-effects in sarilumab-treated RA patients [257,258]. Severity of neutropenia was shown to be dosage-dependent, while there was no relationship between the grade of neutropenia and the frequency of infections [257].

Apart from tocilizumab and sarilumab, other IL-6 neutralizing antibodies are not yet approved for RA treatment, but can be used for treating multicentric Castleman’s disease. However, in vivo studies have indicated the potential of IL-6 neutralizing antibodies for RA treatment [259,260]. RA patients treated with sirukumab had significantly higher ACR20/50 (ACR20: 71.4% for sirukumab vs. 17.6 for placebo control, ACR50: 28.6% vs. 5.9%) and DAS28-CRP response rates (2.1 vs. 0.6), as well as improvement in fatigue scores and depressive symptoms [259,260].

#### 6.3.3. Inhibitors of Co-Stimulation

Abatacept is currently the first member of a new class of biological agents suppressing the induction of inflammation upstream of the pro-inflammatory signaling cascade. Abatacept is a chimeric molecule consisting of the extracellular domain of the co-inhibitory molecule CTLA-4 fused to the Fc portion of a human IgG1 antibody [261]. By neutralizing binding of the CTLA-4 part to either CD80 or CD86 on the surface of activated APCs, abatacept prevents CD80/86-mediated transmission of co-stimulatory signals from APCs to T cells and therefore subsequent T cell activation [262]. Abatacept was approved for the treatment of adults with moderate-to-severe active RA that have either insufficient responses or intolerances to other DMARDs or TNF-α inhibitors [263].

Mechanistically, Okada and colleagues showed abatacept to inhibit osteoclast differentiation, reduce the expression of nuclear factor of activated T cells c 1(NFATc1), and suppress calcium oscillations in bone marrow-derived macrophages in vitro in an FcyR-dependent manner [264]. In fibroblast-like synoviocytes, abatacept treatment reduced levels of MMP1, MMP3, and MMP15 by 50%–60%, while also inhibiting cell migration in a MAPK-dependent way [265].

In addition, human B cells, which can also act as APCS, were shown to be a direct target of abatacept where the drug reduced both surface CD80 and CD86 expression by dynamin-dependent internalization as well as the formation of memory B cells without generally affecting B cell development [266]. As suggested by Lorenzetti and colleagues, reduced surface expression of the co-stimulatory molecules CD80 and CD86 on B cells may impair their ability to provide co-stimulation to T cells as well as the selection and maintenance of autoreactive memory- and plasma cells [266]. In line with this, long-term treatment with abatacept was repeatedly shown to reduce autoantibody levels in RA patients by 50%–90% [266,267]. Therefore, the anti-inflammatory effects of abatacept were more pronounced in RA patients with higher levels of both ACPAs and RF autoantibodies [268,269].

Clinically, abatacept was shown to significantly improve ACR20, -50, and -70 values in comparison to placebo treatment [270]. In the same study, 17.1% of patients treated with abatacept reached low levels of disease activity, and 10% were able to achieve complete remission compared to either 3.1% or 0.8% in the placebo control group, respectively [270]. In the AGREE trial (Abatacept study to Gauge Remission and joint damage progression in methotrexate-naive patients with Early Erosive rheumatoid arthritis) combination treatment with abatacept plus methotrexate was shown to be more effective than treatment with methotrexate alone [271]. Here, both 1-year DAS28-CRP remission rates (adjusted mean changes from baseline in DAS28 CRP were -3.22 for abatacept + methotrexate vs. −2.49 for methotrexate alone) and ACR20, −50 (57.4% of patients achieving ACR50 at one year with abatacept + methotrexate vs. 42.3% with methotrexate alone), −70 (42.6% versus 27.3%) response rates as well as other major clinical response rates were significantly higher while radiographic progression rates were significantly lower in the patient collective that received the combination treatment [271]. In another study comparing abatacept monotherapy with combination therapy of abatacept and methotrexate, abatacept showed either higher or at least comparable efficacy (depending on the time point analyzed) [272]. Here, DAS28 CRP-defined remission (DAS28 CRP <2.6 in 60% of patients treated with abatacept plus methotrexate compared to 45.2% for methotrexate only at 12 months), ACR20, -50, -70 responses, tender and swollen joint counts, patient- as well as physician assessments of pain and disease activity, and the clinical parameters CRP and ESR were all improved compared to placebo treatment [272]. These results suggest that abatacept could also be used in monotherapy approaches.

There are some clinical studies that directly compared abatacept with adalimumab (both on background treatment with methotrexate), which indicate some benefits of abatacept over the TNF-α inhibitor [273,274,275]: two AMPLE studies (Abatacept versus Adalimumab Comparison in Biologic-Naïve RA Patients with Background Methotrexate) showed that the overall disease outcome, improvement on tender and swollen joint count (69.4% ± 2.9% improvement in swollen joint count for abatacept vs. 69.3% ± 2.9% for adalimumab), physician’s global assessment (63.6% ± 4.8% for abatacept vs. 62.8% ± 4.7% for adalimumab), CRP levels, pain (mean ± SEM improvements in pain at year 2: 53.7% ± 6.2% for abatacept vs. 38.5% ± 6.1% for adalimumab), fatigue, as well as ability to perform both work and daily activities were comparable between abatacept and adalimumab [273,274]. Moreover, the improvement in ACR50 response rates over 2 years was also similar (44.7% for abatacept vs. 46.6% for adalimumab) [274]. However, there were less discontinuations in the abatacept group compared with adalimumab due to adverse effects (3.8% for abatacept vs. 9.5% for adalimumab), a lower frequency of serious adverse events (1.6% vs. 4.9%) and severe infections (0/12 vs. 9/19 patients), as well as reduced occurrence of injection site reactions (4.1% vs. 10.4%) [274]. Economically, abatacept was suggested to be a cost-effective alternative to adalimumab in patients with higher ACPAs by Alemao and colleagues after analyzing 646 randomized patients treated with either abatacept or adalimumab (both in combination with methotrexate treatment) [275].

Abatacept is generally well tolerated by RA patients with the most frequent side-effects being upper respiratory tract infections, headaches, and nausea [276]. Thus, abatacept is contraindicated in patients with ongoing severe or uncontrolled infections [276]. While infection was the most frequent side-effect of abatacept therapy [277], patients treated with abatacept were shown to have a reduced risk of hospitalized infections as well as severe infusion/injection reactions compared to other biological DMARDs [278]. Of note, co-administration of abatacept and TNF-α inhibitors is not recommended because of the increased risk of severe infections [276]. In contrast, the risk of malignancies induced by abatacept was shown to be not significantly different from other conventional synthetic or biological DMARDs [278]. Immunogenicity of abatacept was shown to be low with only 4.8% of patients developing antibodies to the molecule [276].

#### 6.3.4. B Cell Depleting Antibodies

While, compared to the activation of autoantigen-specific T cells, autoantibodies are probably not the major driving factor in the establishment of RA, elevated levels of autoantibodies such as RF or ACPAs are highly specific for RA and can precede the onset of the disease by many years [104,279]. Furthermore, immune complexes containing RF or ACPAs may lead to the activation of macrophages resulting in an increased production of TNF-α and CXCL8 which are associated with the manifestation of the disease [110]. Thus, several B cell targeting therapies have been investigated in the last years with rituximab being the only one approved by the FDA in 2006 [104]. It is indicated for RA patients with moderate to severe disease who do not respond adequately to treatment with other DMARDs or at least one TNF inhibitor. Finckh et al. revealed that switching to rituximab after initial therapy failure with one TNF inhibitor was significantly better than switching to a second TNF inhibitor (61% of patients treated with rituximab had an improvement in DAS28 of more than 1.2 units compared to 37% with anti-TNF, decrease in DAS28 21.34 with rituximab compared to 20.93 for alternative anti TNF treatments at 6 months) [280]. Clinically, combination treatment of either cyclophosphamide or methotrexate with rituximab was shown to be both safe and effective for the treatment of RA without resulting in an increased predisposition to infections (compared to placebo) or adversely modifying immunoglobulin levels in patients [281].

While rituximab is overall well tolerated by RA patients, side-effects can include infections, infusion reactions, nervous system disorders, gastrointestinal disorders, and development of psoriasis [282].

Rituximab is a chimeric (mouse/human) monoclonal IgG1 antibody with reactivity against the B cell surface molecule CD20 [104,283]. Originally, it was developed for the treatment of B cell malignancies such as non-Hodgkin’s lymphoma [284]. CD20 is a membrane calcium channel expressed during B cell development starting at the pre-B cell level while being absent from either bone marrow stem cells, pre-B cells, or antibody-producing plasma cells [104,279]. In treated patients repeated administration of rituximab effectively depletes CD20 positive B cells via either (1) antibody-dependent cellular cytotoxicity, (2) complement-mediated cytotoxicity, or (3) apoptosis [285]. Consequently, application of rituximab leads to a targeted depletion of B cells in the peripheral blood but only partial depletion of tissue resident B cells [104,279,283].

A recent study revealed that 4 weeks after rituximab treatment a decrease of naïve and unswitched memory B cells as well as CD21^+^CD23^+^IgD^high^IgM^variable^ follicular B cells occurred [280]. Furthermore, rituximab results in a diminished activation of T cells shown by significantly reduced frequencies of CD3^+^CD4^+^CD69^+^ T cells in treated patients [279].

However, B cell depletion by rituximab treatment was shown to be incomplete as both memory B cells and plasma cell precursors can be still detected in more than half of the RA patients after the first rituximab infusion [283]. In addition, tissue resident B cells are not fully depleted by the treatment. Ramwadhdoebe et al. demonstrated an increased frequency of CD21^+^CD23^+^IgD^high^IgM^variable^ follicular B cells in lymph node biopsies of RA patients [279]. Absence of autoantibodies, high DAS scores, or previous failure of other biologics are associated with decreased response to rituximab. Interestingly, low frequency of CD27^+^ memory B cells may predict better clinical responses to rituximab in RA patients [104].

### 6.4. Limitations of DMARD Therapy

**Figure 7:**
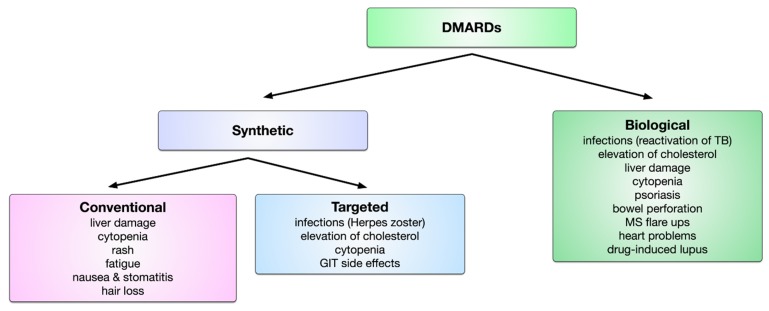
Common side-effects of the available DMARDs. The side-effects are subdivided into the three DMARD classes conventional synthetic, targeted synthetic, and biological. Of note, in addition to common side-effects such as GIT problems, cytopenia, elevation of cholesterol, and liver damage, both targeted synthetic and biological DMARDs result in increased frequencies of infections, likely caused by the inhibition/neutralization of the respective inflammatory mediators and therefore the induction of protective immune responses. TB: tuberculosis.

While DMARDs have repeatedly demonstrated the potential to greatly improve disease symptoms and prevent disease progression in RA patients, they also have considerable side-effects (Figure 7).

In general, conventional synthetic DMARDs, which have been in use for decades at this point, are considered to have less side-effects than either targeted or biological DMARDs [286]. For conventional synthetic DMARDs the profile of side-effects is well characterized including cytopenia, rash, poor tolerability (with nausea, fatigue, hair loss, and stomatitis), as well as in rare cases interstitial lung disease and liver damage (characterized by elevated levels of transaminases) [129].

Due to their overlap in inhibiting pro-inflammatory effector mechanisms, the side-effects associated with the use of targeted synthetic DMARDs and biological DMARDs are similar. Observed side-effects with both classes of DMARDs include increased frequencies of infections, elevated levels of cholesterol, cytopenia (lymphopenia or neutropenia), and gastrointestinal side-effects [129].

In addition, usage of biological DMARDs may result in elevated levels of transaminases, induction or reactivation of autoimmune conditions such as multiple sclerosis and psoriasis, as well as worsening congestive heart failure [129,287,288]. Here, the more frequently observed serious infections include Herpes zoster and other viral infections upon application of targeted synthetic DMARDs [129,289] and the reactivation of latent tuberculosis infections by biological DMARDs (with the exception of rituximab) [290,291]. Therefore, screening for and treatment of latent tuberculosis infections must be conducted before commencing treatment with biological DMARDs.

In addition, the use of biological DMARDs during pregnancy is discussed controversially [1,291]. The idea of treating pregnant women with anti-TNF antibodies was met with skepticism because of their capacity to be transferred from the mother to the unborn child via the umbilical cord. Indeed, in children born to mothers receiving anti-TNF treatment during pregnancy, both adalimumab and infliximab could be detected in the infant’s bloodstream until 12 months of age [292].

In contrast to these findings, results reported by both Mariette and Förger et al. suggested a lack of active transplacental transfer of certolizumab pegol in pregnant women due to its lack of the Fc moiety [293,294]. Analysis of the UCB Pharma database in 2017 including data from 1137 prospectively reported pregnancies did not show evidence of a potential teratogenic effect or an increased risk of fetal death caused by certolizumab, compared to the general population [295]. In line with this, other studies found no correlation between the usage of biological DMARDs and adverse pregnancy outcomes [296] or increased rates of pregnancy-related complications [297].

Moreover, in contrast to conventional synthetic DMARDs therapy, either biological or targeted synthetic DMARDs therapy is associated with a high financial cost, which currently prevents its widespread application in financially restricted settings. Here, the cost of treatment varies between 10,000 Euro (Europe) and 36,000 USD (USA) per year depending on the applied DMARDs and geographical region [7]. Treatment costs can be reduced by the use of biosimilars, if available [298].

## 7. Novel Experimental Strategies in the Treatment of RA

Currently, several strategies to improve the treatment of RA are investigated in experimental animal models. Among others, these include mesenchymal stem cells, application of NOD-, LRR-, and pyrin domain-containing protein 3 (NLRP3) inhibitors, and the targeting of either GM-CSF, GM-CSF receptor, or Toll-like receptor 4.

### 7.1. Mesenchymal Stem Cells

Mesenchymal stem cells (MSCs) are multipotent stromal cells, capable of differentiating into mesenchymal tissues such as bone and cartilage [299], that have also been shown to have immunosuppressive capacities by inhibiting T cell activation in vitro [300,301,302]. Zheng and coauthors stimulated T cells, that were collected from either peripheral blood or synovial fluid of RA patients, with MSCs to explore their therapeutic potential. Here, MSCs significantly suppressed both type II collagen (CII)-stimulated T cell proliferation and T cell activation [303]. In addition, MSCs inhibited IFN-γ and TNF-α secretion from both CD4^+^ and CD8^+^ T cells, which was paralleled by increased production of IL-10 and restored IL-4 secretion [303]. An in vivo study indicated that treatment with MSCs obtained from different sources (bone marrow, umbilical cord, or human exfoliated deciduous teeth) in a mouse model of collagen-induced arthritis significantly improved bone erosions, synovitis, and articular destruction [304]. This improvement of clinical symptoms was paralleled by reduced levels of the pro-inflammatory cytokines TNF-α and IL-1β both in serum and joints [304].

Two initial clinical studies also started to evaluate the safety and potential of umbilical cord blood-derived MSCs on RA patients [305,306]. Here, no adverse events were observed after intravenous infusion of MSCs, while MSC-treated patients showed a tendency towards decreasing Th17 populations and reduced levels of IL-1β, IL-6, IL-8, and TNF-α in peripheral blood [305,306]. However, disease outcome indicators such as CRP, RF, or ESR were not significantly improved either 6 or 12 months after MSCs treatment [306].

### 7.2. Inhibition of NOD-, LRR-, and Pyrin Domain-Containing Protein 3 (NLRP3)

NLRP3 is an intracellular sensor belonging to the family of NOD-like receptors, which can form inflammasome complexes that regulate IL-1β secretion after detection of a wide array of danger signals [307]. Components of the NLRP3 inflammasome have recently been found to be expressed in RA patient’s synovia [308]. These results indicated that inflammasome activation may contribute to pro-inflammatory cytokine secretion in RA patients making inflammasome inhibition a possible therapeutic strategy in the future treatment of RA. In line with this, Guo et al. demonstrated the NLRP3 inflammasome to be strongly activated both in synovia of RA patients and in an in vivo mouse model of collagen-induced arthritis [309]. Here, treatment with MCC950, a selective NLRP3 inhibitor, resulted in both significantly reduced joint inflammation and bone destruction as well as reduced production of IL-1β in vivo [310].

### 7.3. Targeting of GM-CSF and GM-CSF Receptor

GM-CSF is known as a pro-inflammatory cytokine that acts at the interface between innate and adaptive immunity. Several studies have shown both GM-CSF levels to be increased in synovial fluid and plasma of RA patients and the GM-CSF Receptor (GM-CSFR) to be overexpressed in synovial tissue obtained from RA patients [310,311,312]. Cook et al. could show in a mouse model of collagen-induced arthritis, that antibody-mediated neutralization of GM-CSF improved overall disease severity [313,314]. Therefore, several monoclonal antibodies targeting either GM-CSF or GM-CSFR were produced and analyzed. Mavrilimumab (CAM-3001), a monoclonal antibody against the GM-CSFR alpha chain, was shown to improve ACR50 responses compared to placebo in different clinical trials (30.8% for mavrilimumab vs. 12.0% for placebo at week 12; 28.4% vs. 12.3%, at week 24, respectively) [315,316]. Moreover, namilumab (MT203), lenzilumab (KB003), and gimsilumab (MORAb-022) are fully humanized monoclonal IgG1 antibodies targeting GM-CSF [317]. Among these antibodies, namilumab is the only antibody with published data from a phase II clinical trial, indicating that namilumab (dosage 150 mg/every 4 weeks) improved both ACR50 responses (at week 12: 42.9% vs. 14.3%) and DAS28-CRP responses compared to placebo treatment [318].

### 7.4. Toll-Like Receptor 4 (TLR4) Targeting

TLRs play an important role in the initiation and maintenance of both innate and adaptive immune responses. Several endogenous TLR4-ligands such as the small heat shock protein crystalline, B8, or tenascin C are present in the synovial membrane, where they promote joint inflammation with a confirmed role for TLR4 in the pathogenesis of RA [319,320]. In a mouse model of collagen-induced arthritis, TLR4 deficient mice also showed reduced cartilage destruction, lower ACPA production, and decreased IL-17 concentrations compared to wild-type controls [321]. Recently, the first humanized monoclonal antibody targeting TLR4, NI-0101, was tested in a phase II clinical study in patients with RA that had shown inadequate responses to methotrexate. However, the authors reported no significant improvement in the ACR50 response between placebo- and NI-0101-treated groups at week 12 (20.7% in placebo- vs. 14.3% NI-0101-treated patients) [322].

## 8. Treatment Plan of RA

**Figure 8:**
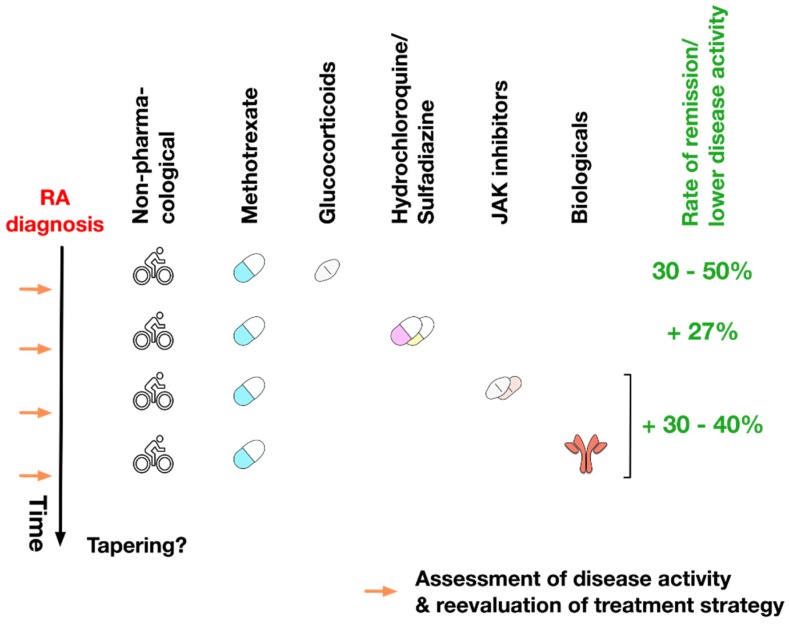
Common treatment plan for the management of RA. Upon initial RA diagnosis (see above) non-pharmacological treatment is provided alongside the application of methotrexate and short-term application of glucocorticoids to reduce joint inflammation, resulting in remission/significantly lower disease activity rates in 30–50% of patients. In non-responsive patients triple therapy consisting of the addition of the conventional synthetic DMARDs hydrochloroquine and sulfadiazine to methotrexate treatment can achieve remission in an additional 10–27% of the patients. Subsequent application of either JAK inhibitors or biological DMARDs can achieve remission/reduced disease activity in an additional 30–40% of RA patients. For optimal treatment it is necessary to constantly reassess both the individual patient’s disease state and efficacy of treatment to make timely adjustments. Once the patient has reached stable remission over at least 6 months, sequential tapering or dose reduction of the administered drugs can be considered in order to reduce treatment-associated side-effects and costs.

Once RA diagnosis is established, a sequential treatment strategy for the management of RA is suggested considering factors such as clinical effects (reduction of inflammation and pain vs. additional prevention of structural damage, see above), profile of side-effects, and costs of therapy (Figure 8).

In a first step non-pharmacological treatment, including physical therapy to maintain joint mobility and patient counselling, is initiated to slow disease progression, which can be maintained during the whole treatment period depending on the status of the individual patient. NSAIDs are usually only used in this early disease stage to either reduce disease symptoms or until the RA diagnosis is established.

First-line RA treatment is usually performed with DMARD monotherapy [2]. Here, non-pharmacological treatment is usually combined with both methotrexate and glucocorticoids for a period of approx. 3–6 months to control inflammation in the newly diagnosed RA patients. Glucocorticoids are usually tapered as soon as possible because their disease-modifying effects are minimal, and their long-term application is associated with multi-systemic side-effects (see above). With this initial treatment regimen 30–50% of RA patients reach either remission or significantly reduced levels of disease activity [7].

In case the treatment target is not reached via methotrexate monotherapy within 3–6 months, other conventional synthetic DMARDs are usually added [2,129]. As reported, compared to monotherapy with methotrexate alone, the addition of hydrochloroquine and sulfasalazine in a triple therapy approach allows disease control in an additional approx. 27% of RA patients [323].

If triple therapy does not achieve the desired outcome, patients (especially with continued high disease activity) should be treated with a combination of methotrexate and either targeted synthetic or biological DMARDs [7]. This drug combination results in disease control in additional 30–40% of RA patients [1].

Of note, biological or targeted synthetic DMARDs should not be considered as first-line treatment since many patients that respond to these drugs were shown to also respond to methotrexate alone. Here, methotrexate has both a lower cost, reduced side-effects, and frequencies of infections compared to the biological or targeted synthetic DMARDs [7].

During the overall treatment process, it is highly important to constantly reassess both the individual patient’s disease state and treatment effectiveness to make timely adjustments. 

While there are no genetic or laboratory markers identified yet that predict the response of individual patients to RA treatment, the presence of either ACPAs or RF antibodies, high disease activity despite treatment with methotrexate, early bone erosion, or cartilage destruction are usually correlated with poor prognosis [1,129]. In contrast to this, early responses to RA treatment (measured in low disease activity after initialization of treatment) correlates with better long-term outcomes [1,7,324].

In patients with persistent remission (usually for at least 6 months), stepwise tapering of RA treatment should be considered to reduce both side-effects (especially for NSAIDs because of toxicity and glucocorticoids because of side-effects) and cost (especially for biological and targeted synthetic DMARDs) of treatment [7,325]. The treatment goal when tapering RA medication is to maintain low disease activity with the lowest medication dose and the fewest number of drugs possible. In this context, tapering of biological DMARDs might be considered, especially if the patient is still treated with methotrexate [129]. If the patient stays in persistent remission after tapering of the biological DMARDs, also tapering of methotrexate can be considered [129].

However, two out of three patients tapering all RA drugs experience disease flare-ups within one year [17,129,325]. Therefore, dose reduction or interval increases between applications should be preferred over complete cessation of therapy [7,326].

## 9. Summary and Conclusion

Although still incurable, both the development of DMARDs and the refinement of non-DMARD therapy have made RA a mostly manageable disease. With the combination of the different available DMARDs many patients are able to reach either full remission or at least significantly reduced disease activity if the disease is diagnosed in an early stage (see above). However, there are still many patients that do not respond to the therapies available so far, demonstrating the need to develop novel drugs/treatment strategies. In this context, markers that allow to predict either therapy outcome or the occurrence of side-effects in individual patients would be highly beneficial for RA treatment. Additionally, the benefits and risks of combinatorial treatment with different DMARDs are yet to be fully understood.

While many drugs can either delay or prevent the onset of RA (methotrexate, sulfasalazine, infliximab, etanercept, abatacept, and rituximab), these positive effects do not seem to prevail once the treatment is stopped [129,240,327,328]. In line with this, the complex network of cell types, cytokines, and chemokines initiating the onset of RA and even more importantly the mechanisms underlying the maintenance of inflammation in the joint need to be further understood in order to improve the existing therapies, identify new targets, and develop new drugs. Finally, despite the great progress in the diagnosis and treatment of RA (see above), the widespread application of (especially biological and targeted synthetic) DMARD therapy is currently still hindered by the associated high costs and frequent occurrence of side-effects (such as for example liver damage, cytopenia or increased frequencies of infections and certain cancers).

## References

[1] Smolen J.S., Aletaha D., McInnes I.B. (2016). Rheumatoid arthritis. Lancet Lond. Engl..

[2] Littlejohn E.A., Monrad S. (2018). Early Diagnosis and Treatment of Rheumatoid Arthritis. Prim. Care: Clin. Off. Pr..

[3] Sacks J.J., Luo Y.-H., Helmick C.G. (2010). Prevalence of specific types of arthritis and other rheumatic conditions in the ambulatory health care system in the United States, 2001–2005. Arthritis Rheum..

[4] Sangha O. (2000). Epidemiology of rheumatic diseases. Rheumatology.

[5] Myasoedova E., Crowson C.S., Kremers H.M., Therneau T.M., Gabriel S.E. (2010). Is the incidence of rheumatoid arthritis rising? Results from Olmsted County, Minnesota, 1955–2007. Arthritis Rheum..

[6] Brzustewicz E., Henc I., Daca A., Szarecka M., Sochocka-Bykowska M., Witkowski J., Bryl E. (2017). Autoantibodies, C-reactive protein, erythrocyte sedimentation rate and serum cytokine profiling in monitoring of early treatment. Cent. Eur. J. Immunol..

[7] Aletaha D., Ramiro S. (2018). Diagnosis and Management of Rheumatoid Arthritis. JAMA.

[8] Deane K.D., Demoruelle M.K., Kelmenson L.B., Kuhn K.A., Norris J.M., Holers V.M. (2017). Genetic and environmental risk factors for rheumatoid arthritis. Best Pr. Res. Clin. Rheumatol..

[9] McGraw W.T., Potempa J., Farley D., Travis J. (1999). Purification, Characterization, and Sequence Analysis of a Potential Virulence Factor from Porphyromonas gingivalis, Peptidylarginine Deiminase. Infect. Immun..

[10] Tan E.M., Smolen J.S. (2016). Historical observations contributing insights on etiopathogenesis of rheumatoid arthritis and role of rheumatoid factor. J. Exp. Med..

[11] Easlick K.A. (1951). An evaluation of the effect of dental foci of infection on health. J. Am. Dent. Assoc..

[12] Wegner N., Wait R., Sroka A., Eick S., Nguyen K.-A., Lundberg K., Kinloch A.J., Culshaw S., Potempa J., Venables P.J. (2010). Peptidylarginine deiminase from Porphyromonas gingivalis citrullinates human fibrinogen and α-enolase: Implications for autoimmunity in rheumatoid arthritis. Arthritis Rheum..

[13] Wilson C., Tiwana H., Ebringer A. (2000). Molecular mimicry between HLA-DR alleles associated with rheumatoid arthritis and Proteus mirabilis as the aetiological basis for autoimmunity. Microbes Infect..

[14] Tiwana H., Wilson C., Alvarez A., Abuknesha R., Bansal S., Ebringer A. (1999). Cross-Reactivity between the Rheumatoid Arthritis-Associated Motif EQKRAA and Structurally Related Sequences Found inProteus mirabilis. Infect. Immun..

[15] Li S., Yu Y., Yue Y., Zhang Z., Su K. (2013). Microbial Infection and Rheumatoid Arthritis. J. Clin. Cell. Immunol..

[16] Ospelt C., Gay S., Klein K. (2017). Epigenetics in the pathogenesis of RA. Semin. Immunopathol..

[17] Aletaha D., Neogi T., Silman A.J., Funovits J., Felson D., Bingham C.O., Birnbaum N.S., Burmester G., Bykerk V., Cohen M.D. (2010). Rheumatoid arthritis classification criteria: An American College of Rheumatology/European League Against Rheumatism collaborative initiative. Ann. Rheum. Dis..

[18] Orozco G., McAllister K., Eyre S. (2011). Genetics of rheumatoid arthritis: GWAS and beyond. Open Access Rheumatol. Res. Rev..

[19] Lenz T.L., Deutsch A., Han B., Hu X., Okada Y., Eyre S., Knapp M., Zhernakova A., Huizinga T.W., Abecasis G. (2015). Widespread non-additive and interaction effects within HLA loci modulate the risk of autoimmune diseases. Nat. Genet..

[20] Zhang Q., Vignali D.A. (2016). Co-stimulatory and Co-inhibitory Pathways in Autoimmunity. Immunology.

[21] Yap H.-Y., Tee S.Z.-Y., Wong M.M.-T., Chow S.-K., Peh S.-C., Teow S.-Y. (2018). Pathogenic Role of Immune Cells in Rheumatoid Arthritis: Implications in Clinical Treatment and Biomarker Development. Cells.

[22] Majorczyk E., Jasek M., Ploski R., Wagner M., Kosior A., Pawlik A., Obojski A., Łuszczek W., Nowak I., Wiśniewski A. (2007). Association of PTPN22 single nucleotide polymorphism with rheumatoid arthritis but not with allergic asthma. Eur. J. Hum. Genet..

[23] Arleevskaya M.I., Kravtsova O.A., Lemerle J., Renaudineau Y., Tsibulkin A.P. (2016). How Rheumatoid Arthritis Can Result from Provocation of the Immune System by Microorganisms and Viruses. Front. Microbiol..

[24] Baka Z., Buzás E., Nagy G. (2009). Rheumatoid arthritis and smoking: Putting the pieces together. Arthritis Res. Ther..

[25] Van Drongelen V., Holoshitz J. (2017). Human Leukocyte Antigen–Disease Associations in Rheumatoid Arthritis. Rheum. Dis. Clin. North. Am..

[26] Viatte S., Plant D., Han B., Fu B., Yarwood A., Thomson W., Symmons D., Worthington J., Young A., Hyrich K.L. (2015). Association of HLA-DRB1 haplotypes with rheumatoid arthritis severity, mortality, and treatment response. JAMA.

[27] Chen J., Li J., Gao H., Wang C., Luo J., Lv Z., Li X. (2012). Comprehensive Evaluation of Different T-Helper Cell Subsets Differentiation and Function in Rheumatoid Arthritis. J. Biomed. Biotechnol..

[28] Coutant F., Miossec P. (2016). Altered dendritic cell functions in autoimmune diseases: Distinct and overlapping profiles. Nat. Rev. Rheumatol..

[29] Jongbloed S.L., Lebre M.C., Fraser A.R., Gracie J.A., Sturrock R.D., Tak P.-P., McInnes I. (2005). Enumeration and phenotypical analysis of distinct dendritic cell subsets in psoriatic arthritis and rheumatoid arthritis. Arthritis Res. Ther..

[30] Page G., Miossec P. (2004). Paired synovium and lymph nodes from rheumatoid arthritis patients differ in dendritic cell and chemokine expression. J. Pathol..

[31] Segura E., Touzot M., Bohineust A., Cappuccio A., Chiocchia G., Hosmalin A., Dalod M., Soumelis V., Amigorena S. (2013). Human Inflammatory Dendritic Cells Induce Th17 Cell Differentiation. Immunity.

[32] Lebre M.C., Jongbloed S.L., Tas S.W., Smeets T.J., McInnes I., Tak P.P. (2008). Rheumatoid Arthritis Synovium Contains Two Subsets of CD83−DC-LAMP− Dendritic Cells with Distinct Cytokine Profiles. Am. J. Pathol..

[33] Tournadre A., Lenief V., Miossec P. (2012). Immature muscle precursors are a source of interferon-β in myositis: Role of Toll-like receptor 3 activation and contribution to HLA class I up-regulation. Arthritis Rheum..

[34] Castañeda-Delgado J.E., Bastian Y., Macias-Segura N., Santiago-Algarra D., Castillo-Ortiz J.D., Alemán-Navarro A.L., Martínez-Tejada P., Enciso-Moreno L., Lira Y.G.-D., Olguín-Calderón D. (2017). Type I Interferon Gene Response Is Increased in Early and Established Rheumatoid Arthritis and Correlates with Autoantibody Production. Front. Immunol..

[35] Cooles F.A., Anderson A., Lendrem D., Norris J., Pratt A., Hilkens C.M.U., Isaacs J.D. (2018). The interferon gene signature is increased in patients with early treatment-naive rheumatoid arthritis and predicts a poorer response to initial therapy. J. Allergy Clin. Immunol..

[36] Page G., Lebecque S., Miossec P. (2002). Anatomic Localization of Immature and Mature Dendritic Cells in an Ectopic Lymphoid Organ: Correlation with Selective Chemokine Expression in Rheumatoid Synovium. J. Immunol..

[37] Chemin K., Gerstner C., Malmström V. (2019). Effector Functions of CD4+ T Cells at the Site of Local Autoimmune Inflammation-Lessons from Rheumatoid Arthritis. Front. Immunol..

[38] Hume D.A. (2015). The Many Alternative Faces of Macrophage Activation. Front. Immunol..

[39] Romagnani S. (2000). T-cell subsets (Th1 versus Th2). Ann. Allergy Asthma Immunol..

[40] Schmidt D., Goronzy J.J., Weyand C.M. (1996). CD4+ CD7- CD28- T cells are expanded in rheumatoid arthritis and are characterized by autoreactivity. J. Clin. Investig..

[41] Fasth A.E.R., Cao D., Van Vollenhoven R., Trollmo C., Malmström V. (2004). CD28nullCD4+ T Cells - Characterization of an Effector Memory T-Cell Population in Patients with Rheumatoid Arthritis. Scand. J. Immunol..

[42] Namekawa T., Wagner U.G., Goronzy J.J., Weyand C.M. (2020). Functional subsets of CD4 T cells in rheumatoid synovitis. Arthritis Rheum..

[43] Griffiths G.M., Alpert S., Lambert E., McGuire J., Weissman I.L. (1992). Perforin and granzyme A expression identifying cytolytic lymphocytes in rheumatoid arthritis. Proc. Natl. Acad. Sci. USA.

[44] Chemin K., Ramsköld D., Diaz-Gallo L.M., Herrath J., Houtman M., Tandre K., Rönnblom L., Catrina A., Malmström V. (2018). EOMES-positive CD4+ T cells are increased in PTPN22 (1858T) risk allele carriers. Eur. J. Immunol..

[45] Wang J., Shan Y., Jiang Z., Feng J., Li C., Ma L., Jiang Y. (2013). High frequencies of activated B cells and T follicular helper cells are correlated with disease activity in patients with new-onset rheumatoid arthritis. Clin. Exp. Immunol..

[46] Ma J., Zhu C., Ma B., Tian J., Baidoo S.E., Mao C., Wu W., Chen J.-G., Tong J., Yang M. (2012). Increased Frequency of Circulating Follicular Helper T Cells in Patients with Rheumatoid Arthritis. Clin. Dev. Immunol..

[47] Zhang Y., Li Y., Lv T.-T., Yin Z.-J., Wang X.-B. (2015). Elevated circulating Th17 and follicular helper CD4 + T cells in patients with rheumatoid arthritis. APMIS.

[48] Gaffen S.L., Jain R., Garg A.V., Cua D.J. (2014). The IL-23–IL-17 immune axis: From mechanisms to therapeutic testing. Nat. Rev. Immunol..

[49] Cascão R., Moura R.A., Perpetuo I., Vieriea-Sousa E., Mourao A.F., Rodrugues A.M., Polido-Pereira J., Queiroz M.V., Rosario H.S., Souto-Carneiro M.M.M. (2020). Identification of a cytokine network sustaining neutrophil and Th17 activation in untreated early rheumatoid arthritis. Arthritis Res. Ther..

[50] Azizi G., Jadidi-Niaragh F., Mirshafiey A. (2013). Th17 Cells in Immunopathogenesis and treatment of rheumatoid arthritis. Int. J. Rheum. Dis..

[51] Kaplan M.J. (2013). Role of neutrophils in systemic autoimmune diseases. Arthritis Res..

[52] Koenders M., Berg W.B.V.D. (2016). Secukinumab for rheumatology: Development and its potential place in therapy. Drug Des. Dev. Ther..

[53] Baker K.F., Isaacs J.D. (2018). Novel therapies for immune-mediated inflammatory diseases: What can we learn from their use in rheumatoid arthritis, spondyloarthritis, systemic lupus erythematosus, psoriasis, Crohn’s disease and ulcerative colitis?. Ann. Rheum. Dis..

[54] Evans H.G., Roostalu U., Walter G.J., Gullick N., Frederiksen K.S., Roberts C., Sumner J., Baeten D.L., Gerwien J.G., Cope A.P. (2014). TNF-α blockade induces IL-10 expression in human CD4+ T cells. Nat. Commun..

[55] Möttönen M., Heikkinen-Eloranta J., Mustonen L., Isomäki P., Luukkainen R., Lassila O. (2005). CD4+ CD25+ T cells with the phenotypic and functional characteristics of regulatory T cells are enriched in the synovial fluid of patients with rheumatoid arthritis. Clin. Exp. Immunol..

[56] Komatsu N., Okamoto K., Sawa S., Nakashima T., Oh-Hora M., Kodama T., Tanaka S.A., Bluestone J., Takayanagi H. (2013). Pathogenic conversion of Foxp3+ T cells into TH17 cells in autoimmune arthritis. Nat. Med..

[57] Wang T., Sun X., Zhao J., Zhang J., Zhu H., Li C., Gao N., Jia Y., Xu D., Huang F.-P. (2014). Regulatory T cells in rheumatoid arthritis showed increased plasticity toward Th17 but retained suppressive function in peripheral blood. Ann. Rheum. Dis..

[58] Nie H., Zheng Y., Li R., Guo T.B., He N., Fang L., Liu X., Xiao L., Chen X., Wan B. (2013). Phosphorylation of FOXP3 controls regulatory T cell function and is inhibited by TNF-α in rheumatoid arthritis. Nat. Med..

[59] Nadkarni S., Mauri C., Ehernstein M.R. (2007). Anti-TNF-alpha therapy induces a distinct regulatory T cell population in patients with rheumatoid arthritis via TGF-beta. J. Exp. Med..

[60] Brennan F.M., McInnes I. (2008). Evidence that cytokines play a role in rheumatoid arthritis. J. Clin. Investig..

[61] Ma H., Xu M., Song Y., Zhang T., Yin H., Yin S. (2019). Interferon-γ facilitated adjuvant-induced arthritis at early stage. Scand. J. Immunol..

[62] Dayer J.M., Beutler B., Cerami A. (1985). Cachectin/tumor necrosis factor stimulates collagenase and prostaglandin E2 production by human synovial cells and dermal fibroblasts. J. Exp. Med..

[63] Bertolini D.R., Nedwin G.E., Bringman T.S., Smith D.D., Mundy G.R. (1986). Stimulation of bone resorption and inhibition of bone formation in vitro by human tumour necrosis factors. Nat..

[64] Marahleh A., Kitaura H., Ohori F., Kishikawa A., Ogawa S., Shen W.-R., Qi J., Noguchi T., Nara Y., Mizoguchi I. (2019). TNF-α Directly Enhances Osteocyte RANKL Expression and Promotes Osteoclast Formation. Front. Immunol..

[65] Lam J., Takeshita S., Barker J.E., Kanagawa O., Ross F.P., Teitelbaum S.L. (2000). TNF-α induces osteoclastogenesis by direct stimulation of macrophages exposed to permissive levels of RANK ligand. J. Clin. Investig..

[66] Kobayashi K., Takahashi N., Jimi E., Udagawa N., Takami M., Kotake S., Nakagawa N., Kinosaki M., Yamaguchi K., Shima N. (2000). Tumor Necrosis Factor α Stimulates Osteoclast Differentiation by a Mechanism Independent of the Odf/Rankl–Rank Interaction. J. Exp. Med..

[67] Azuma Y., Kaji K., Katogi R., Takeshita S., Kudo A. (2000). Tumor Necrosis Factor-α Induces Differentiation of and Bone Resorption by Osteoclasts. J. Boil. Chem..

[68] Fossiez F., Djossou O., Chomarat P., Flores-Romo L., Ait-Yahia S., Maat C., Pin J.J., Garrone P., Garcia E., Saeland S. (1996). T cell interleukin-17 induces stromal cells to produce proinflammatory and hematopoietic cytokines. J. Exp. Med..

[69] Borregaard N. (2010). Neutrophils, from Marrow to Microbes. Immun..

[70] Robert M., Miossec P. (2019). IL-17 in Rheumatoid Arthritis and Precision Medicine: From Synovitis Expression to Circulating Bioactive Levels. Front. Med..

[71] Kotake S., Udagawa N., Takahashi N., Matsuzaki K., Itoh K., Ishiyama S., Saito S., Inoue K., Kamatani N., Gillespie M. (1999). IL-17 in synovial fluids from patients with rheumatoid arthritis is a potent stimulator of osteoclastogenesis. J. Clin. Investig..

[72] Chabaud M., Lubberts E., Joosten L., Berg W.V.D., Miossec P. (2001). IL-17 derived from juxta-articular bone and synovium contributes to joint degradation in rheumatoid arthritis. Arthritis Res..

[73] Van Bezooijen R.L., Papapoulos S.E., Lowik C.W. (2001). Effect of interleukin-17 on nitric oxide production and osteoclastic bone resorption: Is there dependency on nuclear factor-kappaB and receptor activator of nuclear factor kappaB (RANK)/RANK ligand signaling?. Bone.

[74] Chabaud M., Garnero P., Dayer J.-M., Guerne P.-A., Fossiez F., Miossec P. (2000). Contribution of interleukin 17 to synovium matrix destruction in rheumatoid arthritis. Cytokine.

[75] Pickens S.R., Volin M.V., Mandelin A.M., Kolls J.K., Pope R.M., Shahrara S. (2010). IL-17 contributes to angiogenesis in rheumatoid arthritis. J. Immunol..

[76] Ryu S., Lee J.H., Kim S.I. (2005). IL-17 increased the production of vascular endothelial growth factor in rheumatoid arthritis synoviocytes. Clin. Rheumatol..

[77] Xie Y.-D., Jin L., Yu Q.-W. (2007). [The role of IFN-gamma, IL-10, IL-12 and TRAIL in sera and synovium fluids from patients with rheumatoid arthritis]. Chin. J. Cell. Mol. Immunol..

[78] Kokkonen H., Sãderstrãm I., Rocklãv J., Hallmans G., Lejon K., Rantapää-Dahlqvist S., Söderström I., Rocklov J. (2010). Up-regulation of cytokines and chemokines predates the onset of rheumatoid arthritis. Arthritis Rheum..

[79] Steiner G., Tohidast-Akrad M., Witzmann G., Vesely M., Studnicka-Benke A., Gal A., Kunaver M., Zenz P., Smolen J.S. (1999). Cytokine production by synovial T cells in rheumatoid arthritis. Rheumatology.

[80] Morita Y., Yamamura M., Kawashima M., Harada S., Tsuji K., Shibuya K., Maruyama K., Makino H. (1998). Flow cytometric single-cell analysis of cytokine production by CD4+ T cells in synovial tissue and peripheral blood from patients with rheumatoid arthritis. Arthritis Rheum..

[81] Thanapati S., Ganu M., Giri P., Kulkarni S., Sharma M., Babar P., Ganu A., Tripathy A. (2017). Impaired NK cell functionality and increased TNF-α production as biomarkers of chronic chikungunya arthritis and rheumatoid arthritis. Hum. Immunol..

[82] Olalekan S.A., Cao Y., Hamel K.M., Finnegan A. (2015). B cells expressing IFN-γ suppress Treg-cell differentiation and promote autoimmune experimental arthritis. Eur. J. Immunol..

[83] Karonitsch T., Von Dalwigk K., Steiner C.W., Blüml S., Steiner G., Kiener H.P., Ramiro S., Aringer M., Steiner G. (2012). Interferon signals and monocytic sensitization of the interferon-γ signaling pathway in the peripheral blood of patients with rheumatoid arthritis. Arthritis Rheum..

[84] Bach E.A., Aguet M., Schreiber R.D. (1997). THE IFNγ RECEPTOR: A Paradigm for Cytokine Receptor Signaling. Annu. Rev. Immunol..

[85] Schreiber R.D.A., Farrar M., Farrar M., Hershey G.K., Fernandez-Luna J. (1992). The structure and function of interferon-gamma receptors. Int. J. Immunopharmacol..

[86] Tang M., Tian L., Luo G., Yu X. (2018). Interferon-Gamma-Mediated Osteoimmunology. Front. Immunol..

[87] Kwak H.B., Ha H., Kim H.N., Lee J.H., Kim H.S., Lee S., Kim H.M., Kim J.Y., Kim H.H., Song Y.W. (2008). Reciprocal cross-talk between RANKL and interferon-gamma-inducible protein 10 is responsible for bone-erosive experimental arthritis. Arthritis Rheum..

[88] Luster A.D., Ravetch J.V. (1987). Biochemical characterization of a gamma interferon-inducible cytokine (IP-10). J. Exp. Med..

[89] Kim E.Y., Moudgil K.D. (2017). Immunomodulation of autoimmune arthritis by pro-inflammatory cytokines. Cytokine.

[90] Fuller K., Wong B., Fox S., Choi Y., Chambers T. (1998). TRANCE Is Necessary and Sufficient for Osteoblast-mediated Activation of Bone Resorption in Osteoclasts. J. Exp. Med..

[91] Okamoto K., Takayanagi H. (2011). Regulation of bone by the adaptive immune system in arthritis. Arthritis Res..

[92] Yeo L., Schmultz K., Toellner K., Salmon M., Filer A.D., Buckley C., Raza K., Scheel-Toellnew D. (2011). Cytokine mRNA profiling identifies B cells as a major source of RANKL in rheumatoid arthritis. Ann. Rheum. Dis..

[93] Jung S.M., Kim K.W., Yang C.-W., Park S.-H., Ju J.H. (2014). Cytokine-Mediated Bone Destruction in Rheumatoid Arthritis. J. Immunol. Res..

[94] Pettit A., Ji H., Von Stechow D., Müller R., Goldring S.R., Choi Y., Benoist C., Gravallese E.M. (2001). TRANCE/RANKL Knockout Mice Are Protected from Bone Erosion in a Serum Transfer Model of Arthritis. Am. J. Pathol..

[95] Goh F.G., Midwood K.S. (2011). Intrinsic danger: Activation of Toll-like receptors in rheumatoid arthritis. Rheumatol..

[96] Chen Z., Bozec A., Ramming A., Schett G. (2018). Anti-inflammatory and immune-regulatory cytokines in rheumatoid arthritis. Nat. Rev. Rheumatol..

[97] Reboul P., Pelletier J.P., Tardif G., Cloutier J.M., Martel-Pelletier J. (1996). The new collagenase, collagenase-3, is expressed and synthesized by human chondrocytes but not by synoviocytes. A role in osteoarthritis. J. Clin. Investig..

[98] Borden P., Solymar D., Sucharczuk A., Lindman B.R., Cannon P., Heller R.A. (1996). Cytokine Control of Interstitial Collagenase and Collagenase-3 Gene Expression in Human Chondrocytes. J. Boil. Chem..

[99] Redlich K., Smolen J.S. (2012). Inflammatory bone loss: Pathogenesis and therapeutic intervention. Nat. Rev. Drug Discov..

[100] Lefèvre S., Knedla A., Tennie C., Kampmann A., Wunrau C., Dinser R., Korb A., Schnäker E.-M., Tarner I.H., Robbins P.D. (2009). Synovial fibroblasts spread rheumatoid arthritis to unaffected joints. Nat. Med..

[101] Smolen J.S., Aletaha D., Koeller M., Weisman M.H., Emery P. (2007). New therapies for treatment of rheumatoid arthritis. Lancet Lond. Engl..

[102] McInnes I., Schett G. (2011). The Pathogenesis of Rheumatoid Arthritis. N. Engl. J. Med..

[103] Holers V.M., Banda N.K. (2018). Complement in the Initiation and Evolution of Rheumatoid Arthritis. Front. Immunol..

[104] Scher J.U. (2012). B-cell therapies for rheumatoid arthritis. Bull. Nyu Hosp. Jt. Dis..

[105] Nishimura K. (2007). Meta-analysis: Diagnostic Accuracy of Anti–Cyclic Citrullinated Peptide Antibody and Rheumatoid Factor for Rheumatoid Arthritis. Ann. Intern. Med..

[106] Ingegnoli F., Castelli R., Gualtierotti R. (2013). Rheumatoid Factors: Clinical Applications. Dis. Markers.

[107] Steiner G. (2007). Auto-antibodies and autoreactive T-cells in rheumatoid arthritis: Pathogenetic players and diagnostic tools. Clin. Rev. Allergy Immunol..

[108] Wegner N., Lundberg K., Kinloch A.J., Fisher B., Malmström V., Feldmann M., Venables P.J. (2010). Autoimmunity to specific citrullinated proteins gives the first clues to the etiology of rheumatoid arthritis. Immunol. Rev..

[109] Aggarwal R., Liao K., Nair R., Ringold S., Costenbader K.H. (2009). Anti-citrullinated peptide antibody assays and their role in the diagnosis of rheumatoid arthritis. Arthritis Rheum..

[110] Gerlag D.M., Safy M., Maijer K.I., Tang M.W., Tas S.W., Starmans-Kool M.J.F., van Tubergen A., Janssen M., de Hair M., Hansson M. (2019). Tak PP7F1000Prime recommendation of Effects of B-cell directed therapy on the preclinical stage of rheumatoid arthritis: The PRAIRI study. Ann. Rheum. Dis..

[111] Forslind K., Ahlmen M., Eberhardt K., Hafström I., Svensson B. (2004). Prediction of radiological outcome in early rheumatoid arthritis in clinical practice: Role of antibodies to citrullinated peptides (anti-CCP). Ann. Rheum. Dis..

[112] Rönnelid J., Wick M.C., Lampa J., Lindblad S., Nordmark B., Klareskog L., Van Vollenhoven R.F. (2005). Longitudinal analysis of citrullinated protein/peptide antibodies (anti-CP) during 5 year follow up in early rheumatoid arthritis: Anti-CP status predicts worse disease activity and greater radiological progression. Ann. Rheum. Dis..

[113] De Rycke L., Peene I., Hoffman I., Kruithof E., Union A., Meheus L., Lebeer K., Wyns B., Vincent C., Mielants H. (2004). Rheumatoid factor and anticitrullinated protein antibodies in rheumatoid arthritis: Diagnostic value, associations with radiological progression rate, and extra-articular manifestations. Ann. Rheum. Dis..

[114] Coutant F. (2019). Pathogenic effects of anti-citrullinated protein antibodies in rheumatoid arthritis – role for glycosylation. Jt. Bone Spine.

[115] Krishnamurthy A., Joshua V., Hensvold A.H., Jin T., Sun M., Vivar N., Ytterberg A., Engström M., Fernandes-Cerqueira C., Amara K. (2015). Identification of a novel chemokine-dependent molecular mechanism underlying rheumatoid arthritis-associated autoantibody-mediated bone loss. Ann. Rheum. Dis..

[116] Scherer H.U., Van Der Woude D., Ioan-Facsinay A., El Bannoudi H., Trouw L.A., Wang J., Häupl T., Burmester G.-R., Deelder A.M., Huizinga T.W.J. (2010). Glycan profiling of anti-citrullinated protein antibodies isolated from human serum and synovial fluid. Arthritis Rheum..

[117] Nandakumar K.S., Collin M., Olsén A., Nimmerjahn F., Blom A.M., Ravetch J.V., Holmdahl R. (2007). Endoglycosidase treatment abrogates IgG arthritogenicity: Importance of IgG glycosylation in arthritis. Eur. J. Immunol..

[118] Rombouts Y., Ewing E.A., Van De Stadt L., Selman M.H.J., Trouw L.A., Deelder A.M., Huizinga T.W.J., Wuhrer M., Van Schaardenburg D., Toes R. (2013). Anti-citrullinated protein antibodies acquire a pro-inflammatory Fc glycosylation phenotype prior to the onset of rheumatoid arthritis. Ann. Rheum. Dis..

[119] Ercan A., Cui J., Chatterton D.E.W., Deane K.D., Hazen M.M., Brintnell W., O’Donnell C.I., Derber L.A., Weinblatt M.E., Shadick N.A. (2010). IgG galactosylation aberrancy precedes disease onset, correlates with disease activity and is prevalent in autoantibodies in rheumatoid arthritis. Arthritis Rheum..

[120] Pfeifle R., Rothe T., Ipseiz N., Scherer H.U., Culemann S., Harre U.A., Ackermann J., Seefried M., Kleyer A., Uderhardt S. (2016). Regulation of autoantibody activity by the IL-23–TH17 axis determines the onset of autoimmune disease. Nat. Immunol..

[121] Elshabrawy H.A., Chen Z., Volin M.V., Ravella S., Virupannavar S., Shahrara S. (2015). The pathogenic role of angiogenesis in rheumatoid arthritis. Angiogenesis.

[122] Bartók B., Firestein G.S. (2010). Fibroblast-like synoviocytes: Key effector cells in rheumatoid arthritis. Immunol. Rev..

[123] Fassbender H.G., Simmling-Annefeld M. (1983). The potential aggressiveness of synovial tissue in rheumatoid arthritis. J. Pathol..

[124] Baier A., Meineckel I., Gay S., Pap T. (2003). Apoptosis in rheumatoid arthritis. Curr. Opin. Rheumatol..

[125] Yamanishi Y., Boyle D.L., Green D.R., Keystone E.C., Connor A., Zollman S., Firestein G.S. (2004). p53 tumor suppressor gene mutations in fibroblast-like synoviocytes from erosion synovium and non-erosion synovium in rheumatoid arthritis. Arthritis Res. Ther..

[126] Yamanishi Y., Boyle D.L., Rosengren S., Green D.R., Zvaifler N.J., Firestein G.S. (2002). Regional analysis of p53 mutations in rheumatoid arthritis synovium. Proc. Natl. Acad. Sci. USA.

[127] Cha H.-S., Rosengren S., Boyle D.L., Firestein G.S. (2006). PUMA regulation and proapoptotic effects in fibroblast-like synoviocytes. Arthritis Rheum..

[128] Coutant F., Miossec P. (2020). Evolving concepts of the pathogenesis of rheumatoid arthritis with focus on the early and late stages. Curr. Opin. Rheumatol..

[129] Burmester G., Pope J.E. (2017). Novel treatment strategies in rheumatoid arthritis. Lancet.

[130] D’Agostino M.A., Terslev L., Wakefield R., Østergaard M., Balint P., Naredo E., Iagnocco A., Backhaus M., Grassi W., Emery P. (2016). Novel algorithms for the pragmatic use of ultrasound in the management of patients with rheumatoid arthritis: From diagnosis to remission. Ann. Rheum. Dis..

[131] Prado A.D.D., Staub H.L., Bisi M.C., Da Silveira I.G., Mendonça J.A., Pereira J.P., Fonseca J.E. (2018). Ultrasound and its clinical use in rheumatoid arthritis: Where do we stand?. Adv. Rheumatol..

[132] Zayat A.S., Ellegaard K., Conaghan P.G., Terslev L., Hensor E.M.A., Freeston J., Emery P., Wakefield R.J. (2014). The specificity of ultrasound-detected bone erosions for rheumatoid arthritis. Ann. Rheum. Dis..

[133] Yoshimi R., Hama M., Takase K., Ihata A., Kishimoto D., Terauchi K., Watanabe R., Uehara T., Samukawa S., Ueda A. (2013). Ultrasonography is a potent tool for the prediction of progressive joint destruction during clinical remission of rheumatoid arthritis. Mod. Rheumatol..

[134] Iwamoto T., Ikeda K., Hosokawa J., Yamagata M., Tanaka S., Norimoto A., Sanayama Y., Nakagomi D., Takahashi K., Hirose K. (2014). Prediction of Relapse after Discontinuation of Biologic Agents by Ultrasonographic Assessment in Patients With Rheumatoid Arthritis in Clinical Remission: High Predictive Values of Total Gray-Scale and Power Doppler Scores That Represent Residual Synovial. Arthritis Rheum..

[135] Takase-Minegishi K., Horita N., Kobayashi K., Yoshimi R., Kirino Y., Ohno S., Kaneko T., Nakajima H., Wakefield R.J., Emery P. (2017). Diagnostic test accuracy of ultrasound for synovitis in rheumatoid arthritis: Systematic review and meta-analysis. Rheumatology.

[136] Cohen S., Potter H., Deodhar A., Emery P., Conaghan P.G., Østergaard M. (2011). Extremity magnetic resonance imaging in rheumatoid arthritis: Updated literature review. Arthritis Rheum..

[137] Shrive A.K., Holden D., Myles D.A., Greenhough T.J. (1996). Structure Solution of C-Reactive Proteins: Molecular Replacement With a Twist. Acta Crystallogr. Sect. D Boil. Crystallogr..

[138] Baumann H., Gauldie J. (1994). The acute phase response. Immunol. Today.

[139] Kuta A.E., Baum L. (1986). C-reactive protein is produced by a small number of normal human peripheral blood lymphocytes. J. Exp. Med..

[140] Calabrò P., Chang D.W., Willerson J.T., Yeh E.T. (2005). Release of C-Reactive Protein in Response to Inflammatory Cytokines by Human Adipocytes: Linking Obesity to Vascular Inflammation. J. Am. Coll. Cardiol..

[141] Zhang D., Sun M., Samols D., Kushner I. (1996). STAT3 Participates in Transcriptional Activation of the C-reactive Protein Gene by Interleukin-6. J. Boil. Chem..

[142] Calabrò P., Willerson J.T., Yeh E.T. (2003). Inflammatory Cytokines Stimulated C-Reactive Protein Production by Human Coronary Artery Smooth Muscle Cells. Circ..

[143] Siegel J., Osmand A.P., Wilson M.F., Gewurz H. (1975). Interactions of C-reactive protein with the complement system. II. C-reactive protein-mediated consumption of complement by poly-L-lysine polymers and other polycations. J. Exp. Med..

[144] Mold C., Gewurz H., Du Clos T.W. (1999). Regulation of complement activation by C-reactive protein. Immunopharmacology.

[145] Bharadwaj D., Bharadwaj D.-P., Volzer M., Mold C., Du Clos T.W. (1999). The major receptor for C-reactive protein on leukocytes is fcgamma receptor II. J. Exp. Med..

[146] Lu J., Marnell L.L., Marjon K.D., Mold C., Du Clos T.W., Sun P.D. (2008). Structural recognition and functional activation of FcγR by innate pentraxins. Nature.

[147] Williams T.N., Zhang C.X., Game B.A., He L., Huang Y. (2004). C-reactive protein stimulates MMP-1 expression in U937 histiocytes through Fc[gamma]RII and extracellular signal-regulated kinase pathway: An implication of CRP involvement in plaque destabilization. Arterioscler. Thromb. Vasc. Biol..

[148] Nabata A., Kuroki M., Ueba H., Hashimoto S., Umemoto T., Wada H., Yasu T., Saito M., Momomura S.-I., Kawakami M. (2008). C-reactive protein induces endothelial cell apoptosis and matrix metalloproteinase-9 production in human mononuclear cells: Implications for the destabilization of atherosclerotic plaque. Atherosclerosis.

[149] Devaraj S., Yun J.-M., Duncan-Staley C., Jialal I. (2008). C-reactive protein induces M-CSF release and macrophage proliferation. J. Leukoc. Boil..

[150] Han K.H., Hong K.-H., Park J.-H., Ko J.-S., Kang D.-H., Choi K.-J., Hong M.-K., Park S.-W., Park S.-J. (2004). C-Reactive Protein Promotes Monocyte Chemoattractant Protein-1—Mediated Chemotaxis Through Upregulating CC Chemokine Receptor 2 Expression in Human Monocytes. Circulation.

[151] Kim K.-W., Kim B.M., Moon H.W., See S.H., Kim H.R. (2015). Role of C-reactive protein in osteoclastogenesis in rheumatoid arthritis. Arthritis Res. Ther..

[152] Mallya R.K., De Beer F.C., Berry H., Hamilton E.D., Mace B., Pepys M.B. (1982). Correlation of clinical parameters of disease activity in rheumatoid arthritis with serum concentration of C-reactive protein and erythrocyte sedimentation rate. J. Rheumatol..

[153] Matsuno H., Yudoh K., Nakazawa F., Koizumi F. (2002). Relationship between histological findings and clinical findings in rheumatoid arthritis. Pathol. Int..

[154] Wolfe F. (1997). Comparative usefulness of C-reactive protein and erythrocyte sedimentation rate in patients with rheumatoid arthritis. J. Rheumatol..

[155] Van Leeuwen M., Van Der Heijde D.M., Van Rijswijk M.H., Houtman P.M., Van Riel P.L., Van De Putte L.B., Limburg P.C. (1994). Interrelationship of outcome measures and process variables in early rheumatoid arthritis. A comparison of radiologic damage, physical disability, joint counts, and acute phase reactants. J. Rheumatol..

[156] Rhodes B., Fürnrohr B.G., Vyse T. (2011). C-reactive protein in rheumatology: Biology and genetics. Nat. Rev. Rheumatol..

[157] Jansen L.E., Van Der Horst-Bru I., Van Schaardenburg D., Bezemer P.D., Dijkmans B.A.C. (2001). Predictors of radiographic joint damage in patients with early rheumatoid arthritis. Ann. Rheum. Dis..

[158] Devlin J., Gough A., Huissoon A., Perkins P., Holder R., Reece R., Arthur V., Emery P. (1997). The acute phase and function in early rheumatoid arthritis. C-reactive protein levels correlate with functional outcome. J. Rheumatol..

[159] Isiksacan Z., Elbuken C., Erel O. (2016). A portable microfluidic system for rapid measurement of the erythrocyte sedimentation rate. Lab. A Chip.

[160] Ramsay E.S., Lerman M.A. (2014). How to use the erythrocyte sedimentation rate in paediatrics. Arch. Dis. Child. Educ. Pr. Ed..

[161] Radner H., Neogi T., Smolen J.S., Aletaha D. (2013). Performance of the 2010 ACR/EULAR classification criteria for rheumatoid arthritis: A systematic literature review. Ann. Rheum. Dis..

[162] Sokka T., Kautiainen H., Möttönen T., Hannonen P. (1999). Work disability in rheumatoid arthritis 10 years after the diagnosis. J. Rheumatol..

[163] Wolfe F. (1996). The natural history of rheumatoid arthritis. J. Rheumatol. Suppl..

[164] Fries J. (2000). Current treatment paradigms in rheumatoid arthritis. Rheumatology.

[165] Brune K., Patrignani P. (2015). New insights into the use of currently available non-steroidal anti-inflammatory drugs. J. Pain Res..

[166] Crofford L.J. (2013). Use of NSAIDs in treating patients with arthritis. Arthritis Res. Ther..

[167] Van Everdingen A.A., Jacobs J.W., Van Reesema D.R.S., Bijlsma J.W. (2002). Low-dose prednisone therapy for patients with early active rheumatoid arthritis: Clinical efficacy, disease-modifying properties, and side effects: A randomized, double-blind, placebo-controlled clinical trial. Ann. Intern. Med..

[168] Silverstein F.E., Faich G., Goldstein J.L., Simon L.S., Pincus T., Whelton A., Makuch R., Eisen G., Agrawal N.M., Stenson W.F. (2000). Gastrointestinal Toxicity with Celecoxib vs. Nonsteroidal Anti-inflammatory Drugs for Osteoarthritis and Rheumatoid Arthritis. JAMA.

[169] Cronstein B.N. (2005). Low-Dose Methotrexate: A Mainstay in the Treatment of Rheumatoid Arthritis. Pharmacol. Rev..

[170] Abbasi M., Mousavi M.J., Jamalzehi S., Alimohammadi R., Bezvan M.H., Mohammadi H., Aslani S. (2018). Strategies toward rheumatoid arthritis therapy; the old and the new. J. Cell. Physiol..

[171] Emery P.O., Bingham C., Burmester G.R., Bykerk V.P.E., Furst D., Mariette X., Van Vollenhoven R., Arendt C., Mountian I. (2016). Certolizumab pegol in combination with dose-optimised methotrexate in DMARD-naïve patients with early, active rheumatoid arthritis with poor prognostic factors: 1-year results from C-EARLY, a randomised, double-blind, placebo-controlled phase III study. Ann. Rheum. Dis..

[172] Nam J.L., Villeneuve E., Hensor E.M.A., Conaghan P.G.I., Keen H., Buch M.H., Gough A.K., Green M.J., Helliwell P.S., Keenan A.M. (2013). Remission induction comparing infliximab and high-dose intravenous steroid, followed by treat-to-target: A double-blind, randomised, controlled trial in new-onset, treatment-naive, rheumatoid arthritis (the IDEA study). Ann. Rheum. Dis..

[173] Sethi M.K., O’Dell J.R. (2015). Combination conventional DMARDs compared to biologicals. Curr. Opin. Rheumatol..

[174] Weinblatt M.E. (2013). Methotrexate in Rheumatoid Arthritis: A Quarter Century of Development. Trans. Am. Clin. Clim. Assoc..

[175] Nam J.L., Takase-Minegishi K., Ramiro S., Chatzidionysiou K., Smolen J.S., Van Der Heijde D., Bijlsma J.W., Burmester G.R., Dougados M., Scholte-Voshaar M. (2017). Efficacy of biological disease-modifying antirheumatic drugs: A systematic literature review informing the 2016 update of the EULAR recommendations for the management of rheumatoid arthritis. Ann. Rheum. Dis..

[176] Cronstein B.N., Naime D., Ostad E. (1993). The antiinflammatory mechanism of methotrexate. Increased adenosine release at inflamed sites diminishes leukocyte accumulation in an in vivo model of inflammation. J. Clin. Investig..

[177] Rajagopalan P.T.R., Zhang Z., McCourt L., Dwyer M., Benkovic S.J., Hammes G.G. (2002). Interaction of dihydrofolate reductase with methotrexate: Ensemble and single-molecule kinetics. Proc. Natl. Acad. Sci. USA.

[178] Borchers A.T., Keen C.L., Cheema G.S., Gershwin M.E. (2004). The use of methotrexate in rheumatoid arthritis. Semin. Arthritis Rheum..

[179] Van Ede A.E., Laan R.F.J.M., Rood M.J., Huizinga T.W.J., Van De Laar M.A.F.J., Van Denderen C.J., Westgeest T.A.A., Romme T.C., De Rooij D.-J.R.A.M., Jacobs M.J.M. (2001). Effect of folic or folinic acid supplementation on the toxicity and efficacy of methotrexate in rheumatoid arthritis: A forty-eight-week, multicenter, randomized, double-blind, placebo-controlled study. Arthritis Rheum..

[180] Hawkes J.S., Cleland L.G., Proudman S.M., James M.J. (1994). The effect of methotrexate on ex vivo lipoxygenase metabolism in neutrophils from patients with rheumatoid arthritis. J. Rheumatol..

[181] Phillips D.C., Woollard K., Griffiths H.R. (2003). The anti-inflammatory actions of methotrexate are critically dependent upon the production of reactive oxygen species. Br. J. Pharmacol..

[182] Brody M., Böhm I., Bauer R. (1993). Mechanism of action of methotrexate: Experimental evidence that methotrexate blocks the binding of interleukin 1 beta to the interleukin 1 receptor on target cells. Eur. J. Clin. Chem. Clin. Biochem. J. Forum Eur. Clin. Chem. Soc..

[183] Wennerstrand P., Mårtensson L.-G., Söderhäll S., Zimdahl A., Appell M.L. (2013). Methotrexate binds to recombinant thiopurine S-methyltransferase and inhibits enzyme activity after high-dose infusions in childhood leukaemia. Eur. J. Clin. Pharmacol..

[184] Plosker G.L., Croom K.F. (2005). Sulfasalazine: A review of its use in the management of rheumatoid arthritis. Drugs.

[185] Sousa T., Yadav V., Zann V., Borde A., Abrahamsson B., Basit A.W. (2014). On the Colonic Bacterial Metabolism of Azo-Bonded Prodrugsof 5-Aminosalicylic Acid. J. Pharm. Sci..

[186] Situnayake R.D., McConkey B. (1985). Which component of sulphasalazine is active in rheumatoid arthritis?. Br. Med. J. Clin. Res. Ed..

[187] Kumar P., Banik S. (2013). Pharmacotherapy Options in Rheumatoid Arthritis. Clin. Med. Insights: Arthritis Musculoskelet. Disord..

[188] Felson D., Anderson J.J., Meenan R. (1992). Use of short-term efficacy/toxicity tradeoffs to select second-line drugs in rheumatoid arthritis. A metaanalysis of published clinical trials. Arthritis Rheum..

[189] Weinblatt M.E., Reda D., Henderson W., Giobbie-Hurder A., Williams D., Diani A., Docsa S. (1999). Sulfasalazine treatment for rheumatoid arthritis: A metaanalysis of 15 randomized trials. J. Rheumatol..

[190] Öğrendik M. (2013). Antibiotics for the treatment of rheumatoid arthritis. Int. J. Gen. Med..

[191] Rogler G. (2010). Gastrointestinal and liver adverse effects of drugs used for treating IBD. Best Pr. Res. Clin. Gastroenterol..

[192] Farr M., Scott D.G.I., Bacon P.A. (1986). Side Effect Profile of 200 Patients with Inflammatory Arthritides Treated with Sulphasalazine. Drugs.

[193] Farr M., Tunn E., Crockson A.P., Bacon P.A. (1984). The long term effects of sulphasalazine in the treatment of rheumatoid arthritis and a comparative study with penicillamine. Clin. Rheumatol..

[194] Cildag S., Senturk T. (2017). Sulfasalazine-Related Hypersensitivity Reactions in Patients with Rheumatic Diseases. Jcr: J. Clin. Rheumatol..

[195] Alamanos Y., Voulgari P.V., Drosos A.A. (2006). Incidence and Prevalence of Rheumatoid Arthritis, Based on the 1987 American College of Rheumatology Criteria: A Systematic Review. Semin. Arthritis Rheum..

[196] Fox R.I. (1993). Mechanism of action of hydroxychloroquine as an antirheumatic drug. Semin. Arthritis Rheum..

[197] Kyburz D., Brentano F., Gay S. (2006). Mode of action of hydroxychloroquine in RA—evidence of an inhibitory effect on toll-like receptor signaling. Nat. Clin. Pr. Rheumatol..

[198] Suarez-Almazor M.E., Belseck E., Shea B., Homik J.A., Wells G., Tugwell P. (2000). Antimalarials for treating rheumatoid arthritis. Cochrane Database Syst. Rev..

[199] Shinjo S.K., Júnior O.O.M., Tizziani V.A.P., Morita C., Kochen J.A.L., Takahashi W.Y., Laurindo I.M.M. (2007). Chloroquine-induced bull’s eye maculopathy in rheumatoid arthritis: Related to disease duration?. Clin. Rheumatol..

[200] Finbloom D.S., Silver K., Newsome D.A., Gunkel R. (1985). Comparison of hydroxychloroquine and chloroquine use and the development of retinal toxicity. J. Rheumatol..

[201] Marmor M.F., Carr R.E., Easterbrook M., Farjo A.A., Mieler W.F. (2002). Recommendations on screening for chloroquine and hydroxychloroquine retinopathy: A report by the American Academy of Ophthalmology. Ophthalmology.

[202] Peper S.M., Lew R., Mikuls T., Brophy M., Rybin D., Wu H., O’Dell J. (2017). Rheumatoid Arthritis Treatment After Methotrexate: The Durability of Triple Therapy Versus Etanercept. Arthritis Rheum..

[203] Graudal N., Hubeck-Graudal T., Tarp S., Christensen R., Jurgens G. (2014). Effect of Combination Therapy on Joint Destruction in Rheumatoid Arthritis: A Network Meta-Analysis of Randomized Controlled Trials. PLoS ONE.

[204] Goekoop-Ruiterman Y.P.M., De Vries-Bouwstra J.K., Allaart C.F., Van Zeben D., Kerstens P.J.S.M., Hazes J.M.W., Zwinderman A.H., Ronday H.K., Han K.H., Westedt M.L. (2008). Clinical and radiographic outcomes of four different treatment strategies in patients with early rheumatoid arthritis (the BeSt study): A randomized, controlled trial. Arthritis Rheum..

[205] Van Vollenhoven R.F., Geborek P., Forslind K., Albertsson K., Ernestam S., Petersson I., Chatzidionysiou K., Bratt J. (2012). Conventional combination treatment versus biological treatment in methotrexate-refractory early rheumatoid arthritis: 2 year follow-up of the randomised, non-blinded, parallel-group Swefot trial. Lancet.

[206] Moreland L.W., O’Dell J.R., Paulus H.E., Curtis J.R., Bathon J.M., Clair E.W.S., Bridges S.L., Zhang J., McVie T., Howard G. (2012). A randomized comparative effectiveness study of oral triple therapy versus etanercept plus methotrexate in early aggressive rheumatoid arthritis: The treatment of Early Aggressive Rheumatoid Arthritis Trial. Arthritis Rheum..

[207] O’Dell J.R., Taylor T.H., Brophy M., Warren S.R., Cannella A.C., Kunkel G., Leatherman S., Mikuls T.R., Ahluwalia V., Lew R.A. (2013). Therapies for Active Rheumatoid Arthritis after Methotrexate Failure. N. Engl. J. Med..

[208] Sotoudehmanesh R., Anvari B., Akhlaghi M., Shahraeeni S., Kolahdoozan S. (2010). Methotrexate Hepatotoxicity in Patients with Rheumatoid Arthritis. Middle East. J. Dig. Dis..

[209] Taylor W.J., Korendowych E., Nash P., Helliwell P.S., Choy E., Krueger G.G., Soriano E., McHugh N.J., Rosen C.F. (2008). Drug use and toxicity in psoriatic disease: Focus on methotrexate. J. Rheumatol..

[210] Aithal G.P. (2011). Hepatotoxicity related to antirheumatic drugs. Nat. Rev. Rheumatol..

[211] Cummins L., Katikireddi V.S., Shankaranarayana S., Su K.Y.C., Duggan E., Videm V., Pahau H., Thomas R. (2015). Safety and retention of combination triple disease-modifying anti-rheumatic drugs in new-onset rheumatoid arthritis. Intern. Med. J..

[212] Kotyla P.J. (2018). Are Janus Kinase Inhibitors Superior over Classic Biologic Agents in RA Patients?. BioMed Res. Int..

[213] Damsky W., King B. (2017). JAK inhibitors in dermatology: The promise of a new drug class. J. Am. Acad. Dermatol..

[214] Taylor P.C. (2019). Clinical efficacy of launched JAK inhibitors in rheumatoid arthritis. Rheumatology.

[215] Taylor P.C., Keystone E., Van Der Heijde D., Weinblatt M.E., Morales L.D.C., Gonzaga J.R., Yakushin S., Ishii T., Emoto K., Beattie S. (2017). Baricitinib versus Placebo or Adalimumab in Rheumatoid Arthritis. N. Engl. J. Med..

[216] Winthrop K.L. (2017). The emerging safety profile of JAK inhibitors in rheumatic disease. Nat. Rev. Rheumatol..

[217] Rein P., Mueller R.B. (2017). Treatment with Biologicals in Rheumatoid Arthritis: An Overview. Rheumatol. Ther..

[218] Charles P., Elliott M.J., Davis D., Potter A., Kalden J., Antoni C., Breedveld F.C., Smolen J.S., Eberl G., DeWoody K. (1999). Regulation of cytokines, cytokine inhibitors, and acute-phase proteins following anti-TNF-alpha therapy in rheumatoid arthritis. J. Immunol..

[219] Curtis J.R., Xie F., Chen L., Muntner P., Grijalva C.G., Spettell C., Fernandes J., Mcmahan R.M., Baddley J.W., Saag K.G. (2012). Use of a disease risk score to compare serious infections associated with anti-tumor necrosis factor therapy among high- versus lower-risk rheumatoid arthritis patients. Arthritis Rheum..

[220] Leombruno J., Einarson T.R., Keystone E.C. (2008). The safety of anti-tumour necrosis factor treatments in rheumatoid arthritis: Meta and exposure-adjusted pooled analyses of serious adverse events. Ann. Rheum. Dis..

[221] Dreyer L., Mellemkjaer L., Andersen A.R., Bennett P., Poulsen U.E., Ellingsen T., Hansen T.H., Jensen D.V., Linde L., Lindegaard H.M. (2012). Incidences of overall and site specific cancers in TNF inhibitor treated patients with rheumatoid arthritis and other arthritides—A follow-up study from the DANBIO Registry. Ann. Rheum. Dis..

[222] McKenna M.R., Stobaugh D.J., Deepak P. (2014). Melanoma and non-melanoma skin cancer in inflammatory bowel disease patients following tumor necrosis factor-? Inhibitor monotherapy and in combination with thiopurines: Analysis of the Food and Drug Administration Adverse Event Reporting System. J. Gastrointest. Liver Dis..

[223] Askling J., Fahrbach K., Nordstrom B., Ross S., Schmid C.H., Symmons D. (2010). Cancer risk with tumor necrosis factor alpha (TNF) inhibitors: Meta-analysis of randomized controlled trials of adalimumab, etanercept, and infliximab using patient level data. Pharmacoepidemiol. Drug Saf..

[224] Amari W., Zeringue A., McDonald J.R., Caplan L., Eisen S.A., Ranganathan P. (2011). Risk of non-melanoma skin cancer in a national cohort of veterans with rheumatoid arthritis. Rheumatology.

[225] Wolfe F., Michaud K. (2007). Biologic treatment of rheumatoid arthritis and the risk of malignancy: Analyses from a large US observational study. Arthritis Rheum..

[226] Kim S.C., Pawar A., Desai R.J., Solomon D.H., Gale S., Bao M., Sarsour K., Schneeweiss S. (2019). Risk of malignancy associated with use of tocilizumab versus other biologics in patients with rheumatoid arthritis: A multi-database cohort study. Semin. Arthritis Rheum..

[227] Mercer L.K., Green A.C., Galloway J., Davies R., Lunt M., Dixon W.G., Watson K.D., Symmons D., Hyrich K.L. (2012). The influence of anti-TNF therapy upon incidence of keratinocyte skin cancer in patients with rheumatoid arthritis: Longitudinal results from the British Society for Rheumatology Biologics Register. Ann. Rheum. Dis..

[228] Mariette X., Matucci-Cerinic M., Pavelka K., Taylor P., Van Vollenhoven R., Heatley R., Walsh C., Lawson R., Reynolds A., Emery P. (2011). Malignancies associated with tumour necrosis factor inhibitors in registries and prospective observational studies: A systematic review and meta-analysis. Ann. Rheum. Dis..

[229] Wolfe F., Michaud K. (2007). The effect of methotrexate and anti–tumor necrosis factor therapy on the risk of lymphoma in rheumatoid arthritis in 19,562 patients during 89,710 PERSON-YEARS of observation. Arthritis Rheum..

[230] Furst D.E., Schiff M., Fleischmann R., Strand V.A., Birbara C., Compagnone D.A., Fischkoff S., Chartash E.K. (2003). Adalimumab, a fully human anti tumor necrosis factor-alpha monoclonal antibody, and concomitant standard antirheumatic therapy for the treatment of rheumatoid arthritis: Results of STAR (Safety Trial of Adalimumab in Rheumatoid Arthritis). J. Rheumatol..

[231] Van De Putte L.B.A., Atkins C., Malaise M., Sany J., Russell A.S., Van Riel P.L.C.M., Settas L., Bijlsma J.W., Todesco S., Dougados M. (2004). Efficacy and safety of adalimumab as monotherapy in patients with rheumatoid arthritis for whom previous disease modifying antirheumatic drug treatment has failed. Ann. Rheum. Dis..

[232] Weinblatt M.E., Keystone E., Furst D.E., Moreland L.W., Weisman M.H., Birbara C.A., Teoh L.A., Fischkoff S.A., Chartash E.K. (2003). Adalimumab, a fully human anti–tumor necrosis factor α monoclonal antibody, for the treatment of rheumatoid arthritis in patients taking concomitant methotrexate: The ARMADA trial. Arthritis Rheum..

[233] Keystone E., Kavanaugh A., Sharp J.T., Tannenbaum H., Hua Y., Teoh L.S., Fischkoff S.A., Chartash E.K. (2004). Radiographic, clinical, and functional outcomes of treatment with adalimumab (a human anti-tumor necrosis factor monoclonal antibody) in patients with active rheumatoid arthritis receiving concomitant methotrexate therapy: A randomized, placebo-controlled, 52-week trial. Arthritis Rheum..

[234] Furst D.E., Kavanaugh A., Florentinus S., Kupper H., Karunaratne M., Birbara C.A. (2015). Final 10-year effectiveness and safety results from study DE020: Adalimumab treatment in patients with rheumatoid arthritis and an inadequate response to standard therapy. Rheumatology.

[235] Burmester G., Panaccione R., Gordon K., McIlraith M., Lacerda A. (2013). SAT0130 Long-term safety of adalimumab in patients from global clinical trials in rheumatoid arthritis, juvenile idiopathic arthritis, ankylosing spondylitis, psoriatic arthritis, psoriasis, and crohn’s disease. Ann. Rheum. Dis..

[236] Torrente-Segarra V., Arana A.U., Sanchez-Andrade A., Tovar J., Muñoz Á., Martinez A., Gonzalez J., Fernández M., Vazquez N., Corominas H. (2015). RENACER Study: Assessment of 12-month efficacy and safety of 168 certolizumab-PEGol rheumatoid arthritis treated patients from a Spanish multicenter National database. Mod. Rheumatol..

[237] Moreland L.W., Tindall E.A., Weaver A.L., Ettlinger R.E., Mohler K., Widmer M.B., Blosch C.M., Cohen S., Baumgartner S.W., Schiff M. (1997). Treatment of Rheumatoid Arthritis with a Recombinant Human Tumor Necrosis Factor Receptor (p75)–Fc Fusion Protein. N. Engl. J. Med..

[238] Alldred A. (2001). Etanercept in rheumatoid arthritis. Expert Opin. Pharmacother..

[239] Maid P.J., Xavier R., Real R.M., Pedersen R., Shen Q., Marshall L., Solano G., Borlenghi C.E., Hidalgo R.P. (2018). Incidence of Antidrug Antibodies in Rheumatoid Arthritis Patients From Argentina Treated With Adalimumab, Etanercept, or Infliximab in a Real-World Setting. JCR: J. Clin. Rheumatol..

[240] Moots R.J., Xavier R.M., Mok C.C., Rahman M.U., Tsai W.C., Al-Maini M.H. (2017). The impact of anti-drug antibodies on drug concentrations and clinical outcomes in rheumatoid arthritis patients treated with adalimumab, etanercept, or infliximab: Results from a multinational, real-world clinical practice, non-interventional study. PLoS ONE.

[241] Quistrebert J., Hässler S., Bachelet D., Mbogning C., Musters A., Tak P.P., Wijbrandts C.A., Herenius M., Bergstra S.A., Akdemir G. (2019). Incidence and risk factors for adalimumab and infliximab anti-drug antibodies in rheumatoid arthritis: A European retrospective multicohort analysis. Semin. Arthritis Rheum..

[242] Verstappen S., McCoy M.J., Roberts C., Dale N.E., Hassell A.B., Symmons D. (2009). Beneficial effects of a 3-week course of intramuscular glucocorticoid injections in patients with very early inflammatory polyarthritis: Results of the STIVEA trial. Ann. Rheum. Dis..

[243] Van Schouwenburg P., Rispens T., Wolbink G.J. (2013). Immunogenicity of anti-TNF biologic therapies for rheumatoid arthritis. Nat. Rev. Rheumatol..

[244] Krishna M., Nadler S.G. (2016). Immunogenicity to Biotherapeutics – The Role of Anti-drug Immune Complexes. Front. Immunol..

[245] Korswagen L.A., Bartelds G.M., Krieckaert C.L.M., Turkstra F., Nurmohamed M.T., Van Schaardenburg D., Wijbrandts C., Tak P.P., Lems W.F., Dijkmans B.A.C. (2011). Venous and arterial thromboembolic events in adalimumab-treated patients with antiadalimumab antibodies: A case series and cohort study. Arthritis Rheum..

[246] Mihara M., Ohsugi Y., Kishimoto T. (2011). Tocilizumab, a humanized anti-interleukin-6 receptor antibody, for treatment of rheumatoid arthritis. Open Access Rheumatol. Res. Rev..

[247] Avci A.B., Feist E., Burmester G.R. (2018). Targeting IL-6 or IL-6 Receptor in Rheumatoid Arthritis: What’s the Difference?. BioDrugs Clin. Immunother. Biopharm. Gene Ther..

[248] Scott L.J. (2017). Tocilizumab: A Review in Rheumatoid Arthritis. Drugs.

[249] Dhillon S. (2013). Intravenous Tocilizumab: A Review of Its Use in Adults with Rheumatoid Arthritis. BioDrugs.

[250] Nishimoto N., Terao K., Mima T., Nakahara H., Takagi N., Kakehi T. (2008). Mechanisms and pathologic significances in increase in serum interleukin-6 (IL-6) and soluble IL-6 receptor after administration of an anti–IL-6 receptor antibody, tocilizumab, in patients with rheumatoid arthritis and Castleman disease. Blood.

[251] Ogata A., Hirano T., Hishitani Y., Tanaka T. (2012). Safety and Efficacy of Tocilizumab for the Treatment of Rheumatoid Arthritis. Clin. Med. Insights: Arthritis Musculoskelet. Disord..

[252] Ogata A., Kato Y., Higa S., Yoshizaki K. (2019). IL-6 inhibitor for the treatment of rheumatoid arthritis: A comprehensive review. Mod. Rheumatol..

[253] Rafique A., Martin J., Blome M., Huang T., Ouyang A., Papadopoulos N. (2013). AB0037 Evaluation of the binding kinetics and functional bioassay activity of sarilumab and tocilizumab to the human il-6 receptor (il-6r) alpha. Ann. Rheum. Dis..

[254] Raimondo M.G., Biggioggero M., Crotti C., Becciolini A., Favalli E.G. (2017). Profile of sarilumab and its potential in the treatment of rheumatoid arthritis. Drug Des. Dev. Ther..

[255] Emery P., Rondon J., Parrino J., Lin Y., Pena-Rossi C., Van Hoogstraten H., Graham N.M.H., Liu N., Paccaly A., Wu R. (2019). Safety and tolerability of subcutaneous sarilumab and intravenous tocilizumab in patients with rheumatoid arthritis. Rheumatology.

[256] Liang B., Song Z., Wu B., Gardner D., Shealy D.J., Song X.-Y., Wooley P.H. (2009). Evaluation of anti-IL-6 monoclonal antibody therapy using murine type II collagen-induced arthritis. J. Inflamm..

[257] Huizinga T., Fleischmann R.M., Jasson M., Radin A.R., Van Adelsberg J., Fiore S., Huang X., Yancopoulos G.D., Stahl N., Genovese M.C. (2013). Sarilumab, a fully human monoclonal antibody against IL-6Rα in patients with rheumatoid arthritis and an inadequate response to methotrexate: Efficacy and safety results from the randomised SARIL-RA-MOBILITY Part A trial. Ann. Rheum. Dis..

[258] Genovese M.C., Fleischmann R., Kivitz A.J., Rell-Bakalarska M., Martincova R., Fiore S., Rohane P., Van Hoogstraten H., Garg A., Fan C. (2015). Sarilumab plus Methotrexate in Patients With Active Rheumatoid Arthritis and Inadequate Response to Methotrexate: Results of a Phase III Study. Arthritis Rheumatol..

[259] Smolen J.S., Weinblatt M.E., Sheng S., Zhuang Y., Hsu B. (2014). Sirukumab, a human anti-interleukin-6 monoclonal antibody: A randomised, 2-part (proof-of-concept and dose-finding), phase II study in patients with active rheumatoid arthritis despite methotrexate therapy. Ann. Rheum. Dis..

[260] Sun Y., Wang D., Salvadore G., Hsu B., Curran M., Casper C., Vermeulen J., Kent J.M., Singh J., Drevets W.C. (2017). The effects of interleukin-6 neutralizing antibodies on symptoms of depressed mood and anhedonia in patients with rheumatoid arthritis and multicentric Castleman’s disease. Brain behav. Immun..

[261] Bozec A., Luo Y., Engdahl C., Figueiredo C.P., Bang H., Schett G. (2018). Abatacept blocks anti-citrullinated protein antibody and rheumatoid factor mediated cytokine production in human macrophages in IDO-dependent manner. Arthritis Res..

[262] Maxwell L.J., Singh J. (2010). Abatacept for Rheumatoid Arthritis: A Cochrane Systematic Review. J. Rheumatol..

[263] Peichl P., Alten R., Galeazzi M., Lorenz H.-M., Nüßlein H., Navarro F., Elbez Y., Chartier M., Hackl R., Rauch C. (2019). Abatacept retention and clinical outcomes in Austrian patients with rheumatoid arthritis: Real-world data from the 2-year ACTION study. Wien. Med. Wochenschr..

[264] Okada H., Kajiya H., Omata Y., Matsumoto T., Sato Y., Kobayashi T., Nakamura S., Kaneko Y., Nakamura S., Koyama T. (2019). CTLA4-Ig Directly Inhibits Osteoclastogenesis by Interfering With Intracellular Calcium Oscillations in Bone Marrow Macrophages. J. Bone Miner. Res..

[265] Zou Q.-F., Li L., Han Q.-R., Wang Y.-J., Wang X.-B. (2019). Abatacept alleviates rheumatoid arthritis development by inhibiting migration of fibroblast-like synoviocytes via MAPK pathway. Eur. Rev. Med. Pharmacol. Sci..

[266] Lorenzetti R., Janowska I., Smulski C.R., Frede N., Henneberger N., Walter L., Schleyer M.-T., Hüppe J.M., Staniek J., Salzer U. (2019). Abatacept modulates CD80 and CD86 expression and memory formation in human B-cells. J. Autoimmun..

[267] Scarsi M., Paolini L., Ricotta D., Pedrini A., Piantoni S., Caimi L., Tincani A., Airò P. (2014). Abatacept Reduces Levels of Switched Memory B Cells, Autoantibodies, and Immunoglobulins in Patients with Rheumatoid Arthritis. J. Rheumatol..

[268] Sokolove J., Schiff M., Fleischmann R., Weinblatt  M.E., Connolly S.E., Johnsen A., Zhu J., Maldonado M.A., Patel S., Robinson W.H. (2015). Robinson, Impact of baseline anti-cyclic citrullinated peptide-2 antibody concentration on efficacy outcomes following treatment with subcutaneous abatacept or adalimumab: 2-year results from the AMPLE trial. Ann. Rheum. Dis..

[269] Alten R., Nüßlein H.G., Mariette X., Galeazzi M., Lorenz H.-M., Cantagrel A., Chartier M., Poncet C., Rauch C., Le Bars M. (2017). Baseline autoantibodies preferentially impact abatacept efficacy in patients with rheumatoid arthritis who are biologic naïve: 6-month results from a real-world, international, prospective study. Rmd Open.

[270] Genovese M.C., Tena C.P., Covarrubias A., Leon G., Mysler E., Keiserman M., Valente R., Nash P., Simon-Campos J.A., Box J. (2014). Subcutaneous Abatacept for the Treatment of Rheumatoid Arthritis: Longterm Data from the ACQUIRE Trial. J. Rheumatol..

[271] Westhovens R., Robles M., Ximenes A.C., Nayiager S., Wollenhaupt J., Durez P., Gomez-Reino J., Grassi W., Haraoui B., Shergy W. (2009). Clinical efficacy and safety of abatacept in methotrexate-naive patients with early rheumatoid arthritis and poor prognostic factors. Ann. Rheum. Dis..

[272] Emery P., Burmester G.R., Bykerk V.P., Combe B.G., Furst D.E., Barré E., Wong D.A., Huizinga T.W.J. (2014). Evaluating drug-free remission with abatacept in early rheumatoid arthritis: Results from the phase 3b, multicentre, randomised, active-controlled AVERT study of 24 months, with a 12-month, double-blind treatment period. Ann. Rheum. Dis..

[273] Fleischmann R., Weinblatt M.E., Schiff M., Khanna D., Maldonado M.A., Nadkarni A., Furst D.E. (2016). Patient-Reported Outcomes From a Two-Year Head-to-Head Comparison of Subcutaneous Abatacept and Adalimumab for Rheumatoid Arthritis. Arthritis Rheum..

[274] Schiff M., Weinblatt M.E., Valente R., Van Der Heijde D., Citera G., Elegbe A., Maldonado M., Fleischmann R. (2013). Head-to-head comparison of subcutaneous abatacept versus adalimumab for rheumatoid arthritis: Two-year efficacy and safety findings from AMPLE trial. Ann. Rheum. Dis..

[275] Alemao E., Johal S., Al M.J., Molken M.R.-V. (2018). Cost-Effectiveness Analysis of Abatacept Compared with Adalimumab on Background Methotrexate in Biologic-Naive Adult Patients with Rheumatoid Arthritis and Poor Prognosis. Value Heal..

[276] Blair H.A., Deeks E.D. (2017). Abatacept: A Review in Rheumatoid Arthritis. Drugs.

[277] Ogawa N., Ohashi H., Ota Y., Kobori K., Suzuki M., Tsuboi S., Hayakawa M., Goto Y., Karahashi T., Kimoto O. (2019). Multicenter, observational clinical study of abatacept in Japanese patients with rheumatoid arthritis. Immunol. Med..

[278] Ozen G., Pedro S., Schumacher R., Simon T.A., Michaud K. (2019). Safety of abatacept compared with other biologic and conventional synthetic disease-modifying antirheumatic drugs in patients with rheumatoid arthritis: Data from an observational study. Arthritis Res..

[279] Ramwadhdoebe T.H., Van Baarsen L., Boumans M.J.H., Bruijnen S., Safy M., Berger F.H., Semmelink J.F., Van Der Laken C.J., Gerlag D.M., Thurlings R.M. (2019). Effect of rituximab treatment on T and B cell subsets in lymph node biopsies of patients with rheumatoid arthritis. Rheumatology.

[280] Finckh A., Simard J.F., Duryea J., Liang M.H., Huang J., Daneel S., Forster A., Gabay C., Guerne P.-A. (2005). For the Swiss Clinical Quality Management in Rheumatoid Arthritis Project The effectiveness of anti–tumor necrosis factor therapy in preventing progressive radiographic joint damage in rheumatoid arthritis: A population-based study. Arthritis Rheum..

[281] Edwards J.C.W., Szczepanski L., Szchechinski J., Filipowiz-Sosonowska A., Emery P., Close D.R., Stevens R.M., Shaw T. (2004). Efficacy of B-cell-targeted therapy with rituximab in patients with rheumatoid arthritis. N. Engl. J. Med..

[282] Tavakolpour S., AleSaeidi S., Darvishi M., Ghasemiadl M., Darabi-Monadi S., Akhlaghdoust M., Behjati S.E., Jafarieh A. (2019). A comprehensive review of rituximab therapy in rheumatoid arthritis patients. Clin. Rheumatol..

[283] Pollastro S., Klarenbeek P.L., Doorenspleet B.D.C., Esveldt R.E.E., Thurlings R.M., Boumans M.J.H., Gerlag D.M., Tak P.P., Vos K. (2019). Non-response to rituximab therapy in rheumatoid arthritis is associated with incomplete disruption of the B cell receptor repertoire. Ann. Rheum. Dis..

[284] Plosker G.L., Figgitt D.P. (2003). Rituximab: A review of its use in non-Hodgkin’s lymphoma and chronic lymphocytic leukaemia. Drugs.

[285] Pescovitz M.D. (2006). Rituximab, an Anti-CD20 Monoclonal Antibody: History and Mechanism of Action. Arab. Archaeol. Epigr..

[286] Ramiro S., Gaujoux-Viala C., Nam J.L., Smolen J.S., Buch M., Gossec L., Van Der Heijde D., Winthrop K., Landewé R. (2014). Safety of synthetic and biological DMARDs: A systematic literature review informing the 2013 update of the EULAR recommendations for management of rheumatoid arthritis. Ann. Rheum. Dis..

[287] Bongartz T., Sutton A.J., Sweeting M., Buchan I., Matteson E.L., Montori V.M. (2006). Anti-TNF Antibody Therapy in Rheumatoid Arthritis and the Risk of Serious Infections and Malignancies. JAMA.

[288] Mohan N., Edwards E.T., Cupps T.R., Oliverio P.J., Sandberg G., Crayton H., Richert J.R., Siegel J.N. (2001). Demyelination occurring during anti-tumor necrosis factor α therapy for inflammatory arthritides. Arthritis Rheum..

[289] Cohen S.B., Tanaka Y., Mariette X., Curtis J.R., Lee E.B., Nash P., Winthrop K.L., Charles-Schoeman C., Thirunavukkarasu K., Demasi R. (2017). Long-term safety of tofacitinib for the treatment of rheumatoid arthritis up to 8.5 years: Integrated analysis of data from the global clinical trials. Ann. Rheum. Dis..

[290] Tanaka Y., Hirata S., Kubo S., Fukuyo S., Hanami K., Sawamukai N., Nakano K., Nakayamada S., Yamaoka K., Sawamura F. (2013). Discontinuation of adalimumab after achieving remission in patients with established rheumatoid arthritis: 1-year outcome of the HONOR study. Ann. Rheum. Dis..

[291] Wassenberg S., Rau R., Steinfeld P., Zeidler H. (2005). Low-Dose Prednisolone Therapy Study Group Very low-dose prednisolone in early rheumatoid arthritis retards radiographic progression over two years: A multicenter, double-blind, placebo-controlled trial. Arthritis Rheum..

[292] Julsgaard M., Christensen L.A., Gibson P.R., Gearry R.B., Fallingborg J., Hvas C.L., Bibby B.M., Uldbjerg N., Connell W., Rosella O. (2016). Concentrations of Adalimumab and Infliximab in Mothers and Newborns, and Effects on Infection. Gastroenterology.

[293] Förger F., Zbinden A., Villiger P.M. (2016). Certolizumab treatment during late pregnancy in patients with rheumatic diseases: Low drug levels in cord blood but possible risk for maternal infections. A case series of 13 patients. Jt. Bone Spine.

[294] Mariette X., Förger F., Abraham B., Flynn A.D., Molto A., Flipo R.-M., Van Tubergen A., Shaughnessy L., Simpson J., Teil M. (2017). Lack of placental transfer of certolizumab pegol during pregnancy: Results from CRIB, a prospective, postmarketing, pharmacokinetic study. Ann. Rheum. Dis..

[295] Clowse M.E., Wolf U.C., Förger F., Cush J.J., Golembesky A., Shaughnessy L., De Cuyper D., Mahadevan U. (2015). Pregnancy Outcomes in Subjects Exposed to Certolizumab Pegol. J. Rheumatol..

[296] Shimada H., Kameda T., Kanenishi K., Miyatake N., Nakashima S., Wakiya R., Kato M., Miyagi T., Mansour M.M.F., Hata T. (2019). Effect of biologic disease-modifying anti-rheumatic drugs for patients with rheumatoid arthritis who hope to become mothers. Clin. Rheumatol..

[297] Komaki F., Komaki Y., Micic D., Ido A., Sakuraba A. (2017). Outcome of pregnancy and neonatal complications with anti-tumor necrosis factor-α use in females with immune mediated diseases; a systematic review and meta-analysis. J. Autoimmun..

[298] Manova M., Savova A., Vasileva M., Terezova S., Kamusheva M., Grekova D., Petkova V., Petrova G. (2018). Comparative Price Analysis of Biological Products for Treatment of Rheumatoid Arthritis. Front. Pharmacol..

[299] Klontzas M.E., Kenanidis E.I., Heliotis M., Tsiridis E., Mantalaris A. (2015). Bone and cartilage regeneration with the use of umbilical cord mesenchymal stem cells. Expert Opin. Boil. Ther..

[300] Di Nicola M., Carlo-Stella C., Magni M., Milanesi M., Longoni P.D., Matteucci P., Grisanti S., Gianni A.M. (2002). Human bone marrow stromal cells suppress T-lymphocyte proliferation induced by cellular or nonspecific mitogenic stimuli. Blood.

[301] Krampera M., Glennie S., Dyson J., Scott D., Laylor R., Simpson E., Dazzi F. (2003). Bone marrow mesenchymal stem cells inhibit the response of naive and memory antigen-specific T cells to their cognate peptide. Blood.

[302] Aggarwal S., Pittenger M.F. (2005). Human mesenchymal stem cells modulate allogeneic immune cell responses. Blood.

[303] Zheng Z.H., Li X.Y., Ding J., Jia J.F., Zhu P. (2008). Allogeneic mesenchymal stem cell and mesenchymal stem cell-differentiated chondrocyte suppress the responses of type II collagen-reactive T cells in rheumatoid arthritis. Rheumatology.

[304] Zhang Q., Li Q., Zhu J., Guo H., Zhai Q., Li B., Jin Y., He X., Jin F. (2019). Comparison of therapeutic effects of different mesenchymal stem cells on rheumatoid arthritis in mice. PeerJ.

[305] Park E.H., Lim H.-S., Lee S., Roh K., Seo K.-W., Kang K.-S., Shin K. (2018). Intravenous Infusion of Umbilical Cord Blood-Derived Mesenchymal Stem Cells in Rheumatoid Arthritis: A Phase Ia Clinical Trial. Stem Cells Transl. Med..

[306] Ghoryani M., Shariati-Sarabi Z., Afshari J.T., Ghasemi A., Poursamimi J., Mohammadi M. (2018). Amelioration of clinical symptoms of patients with refractory rheumatoid arthritis following treatment with autologous bone marrow-derived mesenchymal stem cells: A successful clinical trial in Iran. Biomed. Pharmacother..

[307] Swanson K.V., Deng M., Ting J.P.-Y. (2019). The NLRP3 inflammasome: Molecular activation and regulation to therapeutics. Nat. Rev. Immunol..

[308] Kolly L., Busso N., Palmer G., Talabot-Ayer D., Chobaz V., So A. (2010). Expression and function of the NALP3 inflammasome in rheumatoid synovium. Immunology.

[309] Guo C., Fu R., Wang S., Huang Y., Li X., Zhou M., Zhao J., Yang N. (2018). NLRP3 inflammasome activation contributes to the pathogenesis of rheumatoid arthritis. Clin. Exp. Immunol..

[310] Williamson D.J., Begley C.G., Vadas M.A., Metcalf D. (1988). The detection and initial characterization of colony-stimulating factors in synovial fluid. Clin. Exp. Immunol..

[311] Xu W.D., Firestein G.S., Taetle R., Kaushansky K., Zvaifler N.J. (1989). Cytokines in chronic inflammatory arthritis. II. Granulocyte-macrophage colony-stimulating factor in rheumatoid synovial effusions. J. Clin. Investig..

[312] Berenbaum F., Rajzbaum G., Amor B., Toubert A. (1994). Evidence for GM-CSF receptor expression in synovial tissue. An analysis by semi-quantitative polymerase chain reaction on rheumatoid arthritis and osteoarthritis synovial biopsies. Eur. Cytokine Netw..

[313] Cook A.D., Braine E.L., Campbell I.K., Rich M.J., Hamilton J.A. (2001). Blockade of collagen-induced arthritis post-onset by antibody to granulocyte-macrophage colony-stimulating factor (GM-CSF): Requirement for GM-CSF in the effector phase of disease. Arthritis Res..

[314] Plater-Zyberk C., Joosten L.A.B., Helsen M.M.A., Hepp J., Baeuerle P.A., Berg W.B.V.D. (2006). GM-CSF neutralisation suppresses inflammation and protects cartilage in acute streptococcal cell wall arthritis of mice. Ann. Rheum. Dis..

[315] Burmester G., Weinblatt M.E., McInnes I., Porter D., Barbarash O., Vatutin M., Szombati I., Esfandiari E., Sleeman M.A., Kane C. (2012). Efficacy and safety of mavrilimumab in subjects with rheumatoid arthritis. Ann. Rheum. Dis..

[316] Burmester G.R., McInnes I., Kremer J., Miranda P., Korkosz M., Vencovský J., Rubbert-Roth A., Mysler E., Sleeman M.A., Godwood A. (2017). A randomised phase IIb study of mavrilimumab, a novel GM–CSF receptor alpha monoclonal antibody, in the treatment of rheumatoid arthritis. Ann. Rheum. Dis..

[317] Šenolt L. (2019). Emerging therapies in rheumatoid arthritis: Focus on monoclonal antibodies. F1000Research.

[318] Taylor P.C., Saurigny D., Vencovský J., Takeuchi T., Nakamura T., Matsievskaia G., Hunt B., Wagner T., Souberbielle B., for the NEXUS Study Group (2019). Efficacy and safety of namilumab, a human monoclonal antibody against granulocyte-macrophage colony-stimulating factor (GM-CSF) ligand in patients with rheumatoid arthritis (RA) with either an inadequate response to background methotrexate therapy or an inadequate response or intolerance to an anti-TNF (tumour necrosis factor) biologic therapy: A randomized, controlled trial. Arthritis Res..

[319] Roelofs M.F., Boelens W.C., Joosten L., Abdollahi-Roodsaz S., Geurts J., Wunderink L.U., Schreurs B.W., Berg W.B.V.D., Radstake T.R.D.J. (2006). Identification of small heat shock protein B8 (HSP22) as a novel TLR4 ligand and potential involvement in the pathogenesis of rheumatoid arthritis. J. Immunol..

[320] Midwood K.S., Sacre S., Piccinini A.M., Inglis J., Trebaul A., Chan E., Drexler S., Sofat N., Kashiwagi M., Orend G. (2009). Tenascin-C is an endogenous activator of Toll-like receptor 4 that is essential for maintaining inflammation in arthritic joint disease. Nat. Med..

[321] Pierer M., Wagner U., Rossol M., Ibrahim S. (2011). Toll-Like Receptor 4 Is Involved in Inflammatory and Joint Destructive Pathways in Collagen-Induced Arthritis in DBA1J Mice. PLoS ONE.

[322] Monnet E., Choy E.H., McInnes I., Kobakhidze T., De Graaf K., Jacqmin P., Lapeyre G., De Min C. (2019). Efficacy and safety of NI-0101, an anti-toll-like receptor 4 monoclonal antibody, in patients with rheumatoid arthritis after inadequate response to methotrexate: A phase II study. Ann. Rheum. Dis..

[323] Erickson A.R., O’Dell J.R. (2016). Lessons Learned From the RACAT Trial: A Comparison of Rheumatoid Arthritis Therapies. Fed. Pr..

[324] Ducreux J., Durez P., Galant C., Toukap A.N., Eynde B.V.D., Houssiau F.A., Lauwerys B.R. (2013). Global Molecular Effects of Tocilizumab Therapy in Rheumatoid Arthritis Synovium. Arthritis Rheumatol..

[325] Smolen J.S., Szumski A., Koening A.S., Jones T.V., Marchall L. (2018). Predictors of remission with etanercept-methotrexate induction therapy and loss of remission with etanercept maintenance, reduction, or withdrawal in moderately active rheumatoid arthritis: Results of the PRESERVE trial. Arthritis Res. Ther..

[326] Smolen J.S., Nash P., Durez P., Hall S., Ilivanoca E., Irazoque-Palazuelos F., Miranda P., Park M.C., Pavelka K., Pedersen R. (2013). Maintenance, reduction, or withdrawal of etanercept after treatment with etanercept and methotrexate in patients with moderate rheumatoid arthritis (PRESERVE): A randomised controlled trial. Lancet Lond. Engl..

[327] Bos W.H., Dijkmans B.A.C., Boers M., Van De Stadt R.J., Van Schaardenburg D. (2009). Effect of dexamethasone on autoantibody levels and arthritis development in patients with arthralgia: A randomised trial. Ann. Rheum. Dis..

[328] Machold K., Landewé R., Ramiro S., Stamm T.A., Van Der Heijde D., Verpoort K.N., Brickmann K., Vázquez-Mellado J., Karateev D., Breedveld F.C. (2010). The Stop Arthritis Very Early (SAVE) trial, an international multicentre, randomised, double-blind, placebo-controlled trial on glucocorticoids in very early arthritis. Ann. Rheum. Dis..

